# Praziquantel Fifty Years on: A Comprehensive Overview of Its Solid State †

**DOI:** 10.3390/pharmaceutics16010027

**Published:** 2023-12-24

**Authors:** Ilenia D’Abbrunzo, Giuseppe Procida, Beatrice Perissutti

**Affiliations:** Department of Chemical and Pharmaceutical Sciences, University of Trieste, Piazzale Europa 1, 34127 Trieste, Italygprocida@units.it (G.P.)

**Keywords:** praziquantel, solid state, crystalline polymorphs, amorphous forms, hydrates, solvates, cocrystals

## Abstract

This review discusses the entire progress made on the anthelmintic drug praziquantel, focusing on the solid state and, therefore, on anhydrous crystalline polymorphs, amorphous forms, and multicomponent systems (i.e., hydrates, solvates, and cocrystals). Despite having been extensively studied over the last 50 years, new polymorphs and the greater part of their cocrystals have only been identified in the past decade. Progress in crystal engineering science (e.g., the use of mechanochemistry as a solid form screening tool and more strategic structure-based methods), along with the development of analytical techniques, including Synchrotron X-ray analyses, spectroscopy, and microscopy, have furthered the identification of unknown crystal structures of the drug. Also, computational modeling has significantly contributed to the prediction and design of new cocrystals by considering structural conformations and interactions energy. Whilst the insights on praziquantel polymorphs discussed in the present review will give a significant contribution to controlling their formation during manufacturing and drug formulation, the detailed multicomponent forms will help in designing and implementing future praziquantel-based functional materials. The latter will hopefully overcome praziquantel’s numerous drawbacks and exploit its potential in the field of neglected tropical diseases.

## 1. Introduction

In recent years, increasing attention has been given to innovative and affordable pharmacological solutions for many neglected tropical diseases, including schistosomiasis. The importance of such treatment clearly appears when considering that roughly 240 million people are currently affected by schistosomiasis and that over 251 million people required preventive treatments in 2021, as reported by the World Health Organization (WHO) [1]. Schistosomiasis is an acute and chronic parasitic disease caused by blood fluke (trematode worms) of the genus *Schistosoma*, prevalent in areas where adequate sanitation and potable water are precluded [2,3,4]. Indeed, infections mainly take place through infested water, where larval forms of the parasite penetrate the skin [5]. Inside the human body, the immature vermin grow into adult schistosomes that release eggs in several tissues, causing bladder cancer, kidney failure, liver fibrosis, intestinal and urinary diseases, etc. [6].

Currently, praziquantel (PZQ) is the recommended drug against all species of schistosomiasis [7,8]. This drug is included in the WHO Model List of Essential Drugs for the treatment of both adults and children [9,10], and the therapeutic regimen provides a dose of 20 mg/kg three times a day at intervals of 4 to 6 h or a single dose of 40 mg/kg as preventive chemotherapy [6,11]. A high dosage mainly depends on the PZQ classification under BCS class II drugs, thus showing high permeability and low solubility (0.40 mg/mL in water at 25 °C) and bioavailability [12,13,14,15]. Moreover, despite being well tolerated, safe, and low-cost, PZQ exhibits an extensive first-pass metabolism (1–3 h) (with mono and di-hydroxy derivatives being the main metabolites [4,8,16,17]).

PZQ is administered orally (trade name Biltricide^®^ [18]) as a racemate [i.e., (RS)-2-(cyclohexylcarbonyl)-1,2,3,6,7,11b-hexahydro-4H-pyrazin[2,1a]-isoquinolin-4-one], even though the pharmacological activity is given by the (R)-enantiomer [19,20,21,22,23,24]. The inactive (S)-enantiomer is responsible for the bitter taste and side effects (e.g., abdominal pain, drowsiness, fever, nausea, and vomiting) [25,26,27,28].

As mentioned above, PZQ has to be administered in relatively high doses due to its low solubility, and it presents some sides effects linked to the (S)-enantiomer. Therefore, the discovery and identification of alternative or new solid forms of the drug are key to improving its physicochemical and biopharmaceutical properties.

Over 50 years of research on PZQ, several studies have been carried out to enhance its properties by preparing solid dispersions of the drug with povidone [17,29,30,31], sodium starch glycolate [32], mesoporous silica [33], calcium carbonate complexes [34,35], mannitol and Gelucire [36], and clay minerals [37], and by preparing PZQ-β-cyclodextrins systems [38,39,40,41,42,43,44], PZQ-liposomes systems [45], polymeric and solid lipid nanoparticles [46,47,48,49], coground systems [50,51,52,53], fast dispersible granules [54], melt granulation and ultrasonic spray congealing [55] and, more recently, micro and nanocrystals [56,57].

Nowadays, alternative approaches to improving PZQ properties have emerged: (i) PZQ deracemization through the formation of diastereomeric cocrystals; (ii) identification of crystalline polymorphs; (iii) the development of multicomponent systems of the drug (i.e., solvates/hydrates and cocrystals).

In this context, this review aims to give a comprehensive overview of all the solid forms of PZQ (i.e., enantiomers, polymorphs, amorphous systems, solvates/hydrates, and cocrystals) that have been found and isolated over 50 years of research (Table 1).

## 2. (RS)-Praziquantel ((RS)-PZQ) (Form A)

### 2.1. Physical and Chemical Properties

**Figure 1:**
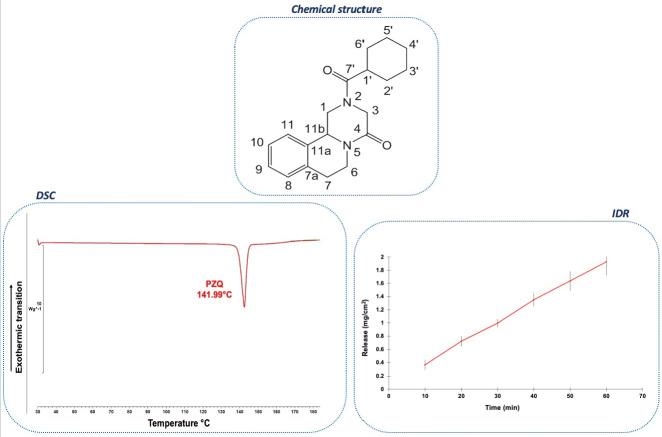
Chemical structure with atom numbering, DSC and IDR of (RS)-PZQ. Adapted from References [58,59].

PZQ is a white crystalline odorless powder well known for its bitter taste [13]. From a chemical point of view, it is a synthetic tetracyclic tetrahydroisoquinoline derivative named (RS)-2-(cyclohexylcarbonyl)-1,2,3,6,7,11b-hexahydro-4H-pyrazin[2,1a]-isoquinolin-4-one whose chemical structure with atom numbering is reported in Figure 1. As evident from Figure 1, PZQ has no hydrogen bond (H-bond) donor groups, while the two amides carbonyl groups can act as H-bond acceptor groups.

The drug is highly hydrophobic, with a LogP of 2.7 [75], sparingly soluble in water at 25 °C (0.40 mg/mL) (between pH 1 and 7.5 [14]), but soluble in ethanol (EtOH) (97 mg/mL) and chloroform (CHF) (567 mg/mL) [12,13,76]. More recently, PZQ solubility was investigated in various solvents at different temperatures and reported as a mole fraction (e.g., at 25 °C, 2.214 × 10^−2^ for acetone (AcT), 4.336 × 10^−2^ for cyclohexanone (CyHXN), 2.291 × 10^−2^ for n-hexane (n-HXN), 2.012 × 10^−2^ for ethyl acetate (EA) and 2.521 × 10^−2^ mole fraction for acetonitrile (ACN)), showing in any case an increase of solubility with the increase of temperature, as typical of an endothermic process [77,78]. PZQ exhibits an intrinsic dissolution rate (IDR) of 31.2 ± 0.6 μg/cm^2^min at 37 °C [59] (Figure 1).

The melting point is reported with a sharp endothermic signal at 141.99 °C with an enthalpy of 91.12 J/g, without any evidence of simultaneous decomposition [13,14] (Figure 1).

### 2.2. Crystal Structure of ((RS)-PZQ) (Form A)

Needle-like single crystals of appropriate size for single-crystal X-ray diffraction (SXRD) analysis were obtained for the first time by Espinosa and coworkers through slow evaporation of a 1:1 solution of (RS)-PZQ and pimelic acid (PIM) in a solvent mixture of AcT/H_2_O [58].

Specifically, (RS)-PZQ, commonly known as Form A and indexed as TELCEU in the Cambridge Structural Database (CSD [79]), crystallizes in the triclinic space group of P-1, showing four crystallographic independent molecules in the asymmetric unit (ASU). Two of the four molecules are disordered (i.e., orientational disorder in the cyclohexyl segment) due to the presence of two rotamers, as attested by dynamic ^1^H NMR measurements [19]; however, all carbonyl groups of PZQ molecules in the unit cell are positioned in the *syn* conformation [58,80,81].

**Figure 2:**
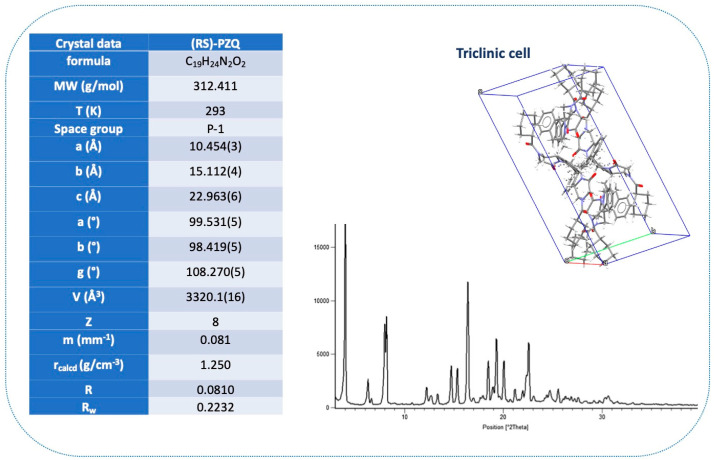
PXRD, crystallographic data, and crystal structure of (RS)-PZQ. Adapted from Reference [58].

Crystallographic data and crystal structure are reported in Figure 2.

### 2.3. Solid-State Characterization Analyses

Besides differential scanning calorimetry (DSC), thermogravimetric analysis (TGA), and powder X-ray diffraction (PXRD) analyses, Fourier transform infrared spectroscopy (FT-IR) and solid-state NMR (SSNMR) are the main solid-state techniques used to understand crystal polymorphism and interactions between PZQ and coformers in all its multicomponent solid forms discovered so far.

Therefore, a brief digression on these two techniques (i.e., (RS)-PZQ assignments in FT-IR and SSNMR spectra) is mandatory and it is presented below.

#### 2.3.1. FT-IR Spectrum

Various FT-IR experimental spectra of (RS)-PZQ were reported in the literature (also with ATR methods) [32,40,48,55,82,83], where some differences and discrepancies were found in the assignments of some vibration bands, especially in the carbonyl group modes, later optimized through calculations based on the Density Functional Theory (DFT) by means of the DMOL3 program [80].

Specifically, (RS)-PZQ shows two main characteristic bands in the range 3028–2852 cm^−1^ assigned to stretching vibrations ν(CH) of CH and CH_2_ groups, with aromatic CH bonds at higher frequencies than the aliphatic ones. The ν(C=O) bands were observed in the range 1736–1627 cm^−1^ with no relative assignment for the two C=O (i.e., heterocyclic carbonyl and cyclohexyl carbonyl) of the drug. This deficiency has been explained considering that both C=O bonds have similar local environments, thus making it difficult to assign each band to one of the two carbonyl groups [32,40,48,55,80,82]. In a later article, Borrego et al. distinguished the two ν(C=O) bands through theoretical calculations. In particular, they suggested that the stretching of twisted C-C=O planes appears at high frequencies (for the heterocyclic C=O group), while the other appears at lower frequencies (for the carbonyl group joined to the cyclohexyl, where both groups are coplanar) [81]. 

Other authors reported bands of stretching vibration ν(C-H) or bending δ(CH) of the C-H bonds and bands of stretching vibration of the C-N bonds (ν(CN)) in the range 1450–1200 cm^−1^ [40,48], a slightly downward shifted compared to those calculated by Borrego and coauthors [80]. 

Spectra of the calculated (DFT) *anti* and *syn* coformers of (RS)-PZQ were simulated and compared to experimental data. In the *syn* coformer, both carbonyl groups are oriented to the COCH_2_N group, and the effect of the local dipole is higher than in *anti*. Therefore, the frequency difference in ν(C=O) between both carbonyl groups is higher in *syn* than in *anti* [80]. The same authors, in a subsequent study, stated that the *anti*-conformation is less polar than the *syn* one and, thus, more stable in an apolar environment [81]. A comparison between the two calculated conformations and the experimental frequencies determined for the carbonyl groups (bands at 1647 and 1624 cm^−1^) allowed the confirmation that (RS)-PZQ Form A exhibits the *syn* conformation, as clearly determined from PZQ molecular packing in the crystal structure [58,59,80,81].

**Figure 3:**
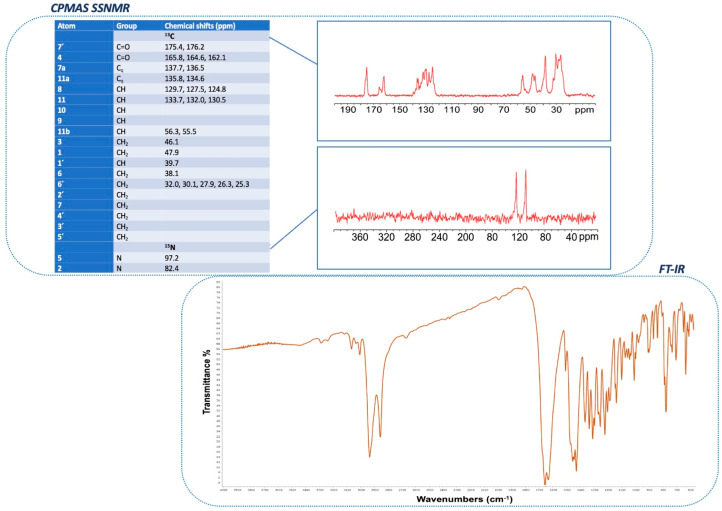
SSNMR and FT-IR spectra of (RS)-PZQ. Adapted from References [58,59].

FT-IR spectrum of (RS)-PZQ is visible in Figure 3.

#### 2.3.2. SSNMR Analysis

The ^13^C and ^15^N CPMAS SSNMR spectra of (RS)-PZQ are reported by Arrúa et al., Costa et al., and Zanolla et al. [30,42,59]. 

The ^13^C CPMAS spectrum is characterized by the splitting of all signals, as apparent from resonance around 164 ppm assigned to carbonyl C4 (see Figure 1 for atom numbering). This splitting is a confirmation of the presence of four crystallographic independent molecules in the unit cell, in accordance with the crystal structure. 

Three regions are brought to light: the aliphatic carbon region between 20 and 60 ppm, the aromatic carbon region between 120 and 135 ppm, and the carbonyl region at 160–175 ppm.

The ^15^N CPMAS spectrum clarifies resonances for N2 and N5 atoms: specifically, the two nitrogens resonate at 82.4 and 97.2 ppm, respectively.

Figure 3 reports experimental ^13^C and ^15^N spectra and chemical shifts (ppm) with specific assignments for all atoms of (RS)-PZQ. 

## 3. PZQ Enantiomers

As briefly discussed in the introduction, PZQ is administered as a racemate ((RS)-PZQ), although it is evident that the schistosomicidal activity relies on the (R)-enantiomer, while the (S)-enantiomer is mainly responsible for the bitter taste and side effects [23,24,28]. Moreover, there is evidence in the literature that the administration of the isolated (R)-eutomer can reduce adverse effects and, thus, the dosage needed [84]. For this reason, the resolution of the PZQ racemic compound has been and still is a justified approach. 

Enantiomerically pure (S)-(+)-PZQ and (R)-(–)-PZQ were isolated as hemihydrates ((S)-PZQ-HH [25] and (R)-PZQ-HH) [26] through an integrated method of chromatography, used to enantio-enrich the starting solution, and subsequent crystallization from a methanol/water (MeOH/H_2_O) mixture. These two hydrated forms allow, for the first time, the solving of the crystal structures of the two pure enantiomers (indexed in the CSD as SIGBUG and SIGBUG01, respectively). They both crystallize in the monoclinic space group of C2, showing four molecules in the unit cell linked through H-bonds and just one independent molecule in the ASU. The two crystal structures indicated that the conformation of the two enantiomers in the unit cell is the *anti*-conformation, instead of the *syn* observed for racemic (RS)-PZQ, since the H_2_O molecule maintains H-bonds with the carbonyl groups from different molecules acting as bridging H-bonds [34,80,81]. The melting point reported for the two enantiomers is roughly 110 °C, thus implying advantages in terms of solubility when compared to racemic (RS)-PZQ [76,83].

In 2013, Cedillo and coauthors [85] used praziquanamine for the isolation of an enantiomerically pure PZQ derivative ((S)-(+)-cis-4-benzyloxy-PZQ) as an intermediate for the synthesis of (S)-(+)-cis-4-hydroxy-PZQ, one of the main metabolites of PZQ [16]. The crystal structure of (S)-(+)-cis-4-benzyloxy-PZQ was deposited in the CSD with the CCDC reference code RIMBIA. 

One year later, the same scientific group used an alternative method for the isolation of the (R)-eutomer of PZQ. The method consisted of the formation of a pair of diastereoisomers derived from (S)-(+)-naproxen (i.e., (R)-(–)-PZQ-(S)-naproxen and (S)-(+)-PZQ-(S)-naproxen) which were easily separated by flash column chromatography, with a subsequent acidic hydrolysis and acylation of the wanted diastereoisomer (i.e., ((R)-(–)-PZQ-(S)-naproxen, indexed as LIVDOL in the CSD) with cyclohexanecarbonyl chloride to provide enantiomerically pure (R)-PZQ as a monohydrate ((R)-PZQ-MH) [19].

In the same work, starting from (R)-PZQ-MH and through a four-step sequence synthesis, it was also possible to obtain enantiomerically pure (R)-PZQ derivatives (i.e., (R)-(–)-trans-4-hydroxy-PZQ-MH); (R)-(–)-trans-4-benzyloxy-PZQ); (R)-(–)-cis-4-benzyloxy-PZQ). Specifically, crystal structures of (R)-PZQ-MH, (R)-(–)-trans-4-hydroxy-PZQ-MH, (R)-(–)-trans-4-benzyloxy-PZQ, and (R)-(–)-cis-4-benzyloxy-PZQ were solved and indexed in the CSD as LIVFED, LIVFIH, LIVDUR, and LIVFAZ, respectively. 

Interestingly, PZQ molecules of (R)-PZQ-MH exhibit the same *anti*-conformation observed for the two previously described enantiomeric hemihydrates (i.e., SIGBUG and SIGBUG01), in opposition to the *syn* observed for racemic (RS)-PZQ.

A 2015 paper reported the chiral resolution of (RS)-PZQ through the formation of diastereomeric co-crystal pairs with enantiomerically pure L-malic acid (L-MAL) as the coformer [68]. The authors initially performed liquid-assisted grinding (LAG) with 10 μL of AcT using a 1:1 stoichiometric mixture of (RS)-PZQ and the cocrystal coformer for 30 min at 25 Hz. As PXRD evidenced a new phase corresponding neither to pure (RS)-PZQ and L-MAL nor to pure PZQ enantiomers, they subsequently synthetized (R)-PZQ and (S)-PZQ as hemihydrates; then, they subjected the two pure enantiomeric compounds to LAG in the presence of L-MAL. They obtained two cocrystals (i.e., (R)-PZQ/L-MAL and (S)-PZQ/L-MAL) that were nothing but the sum of the products obtained from the initial analogous grinding experiment with the (RS)-PZQ racemate, thus indicating that the first novel phase obtained comprises a mixture of the diastereomeric cocrystals (R)-PZQ/L-MAL and (S)-PZQ/L-MAL. The crystal structures of these two cocrystals were solved, showing that (R)-PZQ/L-MAL and (S)-PZQ/L-MAL (indexed in the CSD as CUZPIY and CUZPEU, respectively) crystallize in the same space group (P2_1_) and have similar unit cell parameters. They both show H-bonds between the amide group of PZQ and the carboxylic acid groups of L-MAL, displaying the *anti*-conformation of PZQ carbonyl groups; however, (R)-PZQ/L-MAL revealed stronger intermolecular interactions, confirmed by DSC analysis, through which a higher melting point was observed when compared to (S)-PZQ/L-MAL. Additionally, comparative solubility experiments in various solvents showed significant variations in the solubility of the two diastereomeric cocrystals when using AcT or EA. Based on these results, the authors decided to chiral resolve racemic (RS)-PZQ via fractional crystallization from AcT or EA in the presence of L-MAL, followed by a second step of phase decomposition of the (R)-PZQ/L-MAL cocrystal by treatment with H_2_O to obtain (R)-PZQ-HH. Finally, the authors performed IDR on the (R)-PZQ/L-MAL cocrystal by comparing it to pristine (R)-PZQ-HH and (RS)-PZQ. Interestingly, the (R)-PZQ/L-MAL cocrystal exhibited a significantly greater IDR constant than (R)-PZQ-HH and (RS)-PZQ (a 6.1- and 12.5-fold increase, respectively).

In 2021, Valenti and coworkers developed an efficient deracemization method by coupling incompatible racemization and crystallization processes [86]. They firstly identified, through a screening among 30 crystalline derivatives, a derivative (i.e., PZQ + pivaloyl derivative) that crystallizes as a conglomerate and then performed racemization. Racemization occurred via reversible hydrogenation over a palladium on carbon (Pd/C) packed column at 130 °C, whereas the deracemization was obtained by alternating crystal growth/dissolution steps with temperature cycling between 5–15 °C. These incompatible processes were combined by means of a flow system through which a complete deracemization of the solid phase into the desired (R)-enantiomer was obtained.

## 4. PZQ Crystalline Polymorphs

Structural and crystal polymorphism is a known property of the solid state. IUPAC refers to a polymorph as a solid crystalline phase with the same chemical composition of another phase with a different crystal structure [87]. In fact, polymorphs have different relative intermolecular and/or interatomic distances and, therefore, structural differences that are sometimes significant in changing physical and chemical characteristics of a specific drug [88,89,90].

Based on that, polymorphism has recently emerged as one of the valid alternatives to improve some physicochemical properties of pharmaceutical solids, without introducing exogenous atoms or molecules in the API structure.

Although investigations on PZQ started 50 years ago and the compound was approved for the treatment of human schistosomiasis in 1980 [2,91,92], the first polymorphic form of the racemic drug, named Form B, only appeared in the literature in 2018. Previous works have tried to obtain new polymorphs of PZQ by slow evaporation using several solvents (i.e., H_2_O, EtOH, MeOH, 2-propanol (2-PROH), 1,2-ethanediol (1,2-EtdOH), and dimethyl sulfoxide (DMSO)), but the same starting Form A always emerged [93].

Conversely, polymorph B was obtained for the first time by Zanolla et al. by milling the commercial Form A via the neat grinding (NG) method (in the complete absence of solvent) at 20 Hz for 4 h [59]. The new form was chemically identical to the starting material (as proved by HPLC, ^1^H NMR, and polarimetry analyses) but exhibited different physical properties and very favorable biopharmaceutical characteristics.

**Figure 4:**
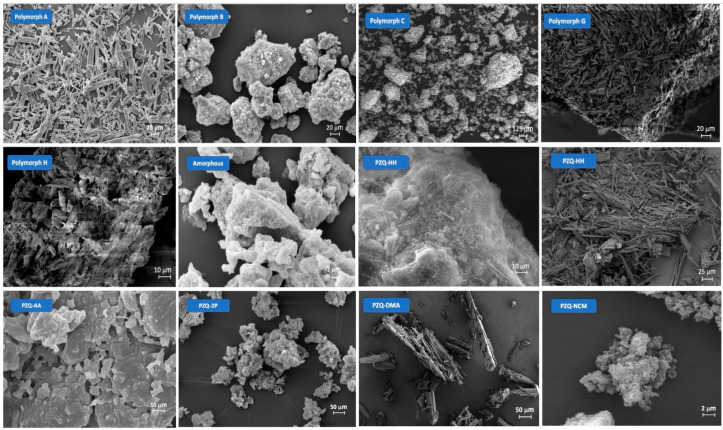
SEM images of polymorph A (500×), polymorph B (1000×), polymorph C (50×), polymorph G (295×), polymorph H (775×), amorphous form (5000×), PZQ-HH (1000×), PZQ-HH from CO_2_ supercritical (500×), PZQ-AA (1500×), PZQ-2P (370×), PZQ-DMA (160×), and PZQ-NCM cocrystal (5000×). Adapted from references [59,60,62,64,65,66,73].

**Figure 5:**
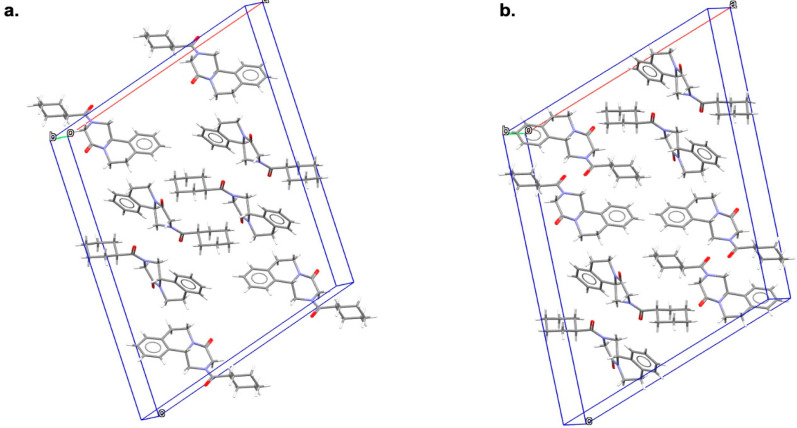
Crystal structures of (**a**) polymorph B and (**b**) polymorph C of PZQ.

Starting from scanning electron microscopy (SEM) images, Form B revealed a very different habitus in comparison to the acicular shape crystals of Form A (see Figure 4): crystals of this new form are agglomerates made of long and very thin whiskers. The thermal behavior of Form B was evaluated through DSC and TGA. In accordance with TGA results which attested to the anhydrous nature of the solid, the DSC curve showed a unique endothermic peak at 112.10 °C, attributable to its melting, without any dehydration event. Moreover, the PXRD pattern of the polymorphic form clearly indicated that the new phase and Form A were different, and the crystal structure of Form B was solved from a powder Synchrotron X-ray diffraction pattern. Form B (indexed in the CSD as TELCEU01) crystallizes in the centrosymmetric monoclinic space group of *C*2/*c*, presenting eight molecules in the unit cell and one crystallographic independent molecule in the ASU (see Figure 5). The ^13^C CPMAS SSNMR spectrum confirmed this finding, as it was characterized by a single set of resonances, instead of all split signals observed for Form A, owing to the four independent molecules. All the peaks observed in both ^13^C and ^15^N CPMAS SSNMR spectra were very sharp and slightly shifted compared to original PZQ, attesting to the formation of a new and crystalline phase. FT-IR spectroscopy clarified the conformation of PZQ carbonyl moieties in the unit cell: Form A shows two signals at 1647 and 1624 cm^−1^ attributable to the stretching of the heterocyclic carbonyl and the carbonyl group joined to the cyclohexyl, respectively [50,58,59,81]. These two peaks nearly overlapped in Form B, showing a frequency difference in the carbonyl vibrations that is lower than Form A and thus confirming the presence of the *anti*-conformation [34], in accordance with the theoretical calculations [80].

Regarding biopharmaceutical properties, polymorph B presented double water solubility and IDR (281.31 ± 8.32 mg/L at 20 °C and 62.2 ± 1.3 μg cm^−2^ min^−1^ at 37 °C, respectively) compared to commercial Form A (140.30 ± 9.26 mg/L at 20 °C and 31.2 ± 0.6 μg cm^−2^ min^−1^ at 37 °C, respectively). Moreover, the in vitro antischistosomal activity against adult *Schistosoma mansoni* was also determined. After 72 h, Form B manifested an IC_50_ of 0.07 μg/mL, comparable to that observed for Form A (0.05 μg/mL). The in vivo anthelmintic activity was also evaluated: Form B and Form A were orally administered as suspension to a total of 66 infected mice after seven weeks post-infection. They both reduced the overall worm burden (WBR) significantly compared to the control mice, and the highest WBR was achieved at 300 mg/kg (WBR of 89% for Form B and WBR of 87% for Form A) [94].

Finally, stability studies revealed that Form B was physically stable under dry conditions at room temperature for at least 12 months.

Later, other different strategies to obtain polymorph B were also discovered. Specifically, Zanolla et al. also reported the possibility of accessing Form B starting from PZQ hemihydrate (PZQ-HH) with different processes: (i) by monitoring PZQ-HH physical stability, it was observed that after 4 months the hemihydrate converted to a polymorphic form of PZQ that was, indeed, Form B; (ii) Form B was likewise obtained by leaving PZQ-HH at 50 °C under vacuum (35 mmHg) overnight; (iii) Form B also unfolded after milling the hemihydrate for 60 min at 25 Hz [64].

Polymorph B was further observed by another scientific group investigating the effect of two factors (i.e., filtration of the prepared solution before cooling and agitation) on cooling crystallization of (RS)-PZQ Form A in the presence of triethylamine (TEA) [62]). Indeed, they demonstrated that cooling crystallization in TEA performed without filtration of the prepared solution before cooling and without agitation gave rise to Form B. In that work, TEA was the only screened solvent that allowed the formation of polymorph C, instead of Form A, and could possibly form other polymorphs.

By exploring the experimental design space around polymorph B formation as a function of two milling parameters (i.e., frequency and milling time) through a rotated Doehlert matrix [95], Zanolla et al. also found another PZQ polymorph, namely Form C [60]. Precisely, the Doehlert matrix was designed using NEMRODW software [96] in which 180 min < x_1_ < 300 min and 15 Hz < x_2_ < 25 Hz were considered as time and frequency constraints, respectively. In the central zone of the hexagonal design space, the presence of Form B was attested, whereas a mixture of starting material/amorphous/various polymorphic forms was observed when using lower mechanochemical energy (i.e., lower frequency and higher milling time). Conversely, four different NG conditions (i.e., 240 min at 23 Hz, 256 min at 25 Hz, 198 min at 24 Hz, and 214 min at 19 Hz) successfully gave rise to a new phase, with NG for 240 min at 23 Hz appearing as the best operating condition, in terms of frequency and milling time, to bring forth the highest crystallinity polymorph C. SEM images showed that Form C exhibited clustered particles, different from the needle-shaped particles of Form A and the little whiskers of Form B (Figure 4). The DSC curve exhibited a unique melting peak at 106.84 °C without any dehydration event or other thermal events (confirmed by TGA) and PXRD pattern, albeit generally like Form B, it showed characteristic reflections through which the crystal structure of the new solid was solved by means of Synchrotron X-ray powder diffraction. Form C (indexed as GOYZOM in the CSD) crystallizes in the centrosymmetric monoclinic space group I2/*c*, hosting eight molecules and showing one crystallographic independent molecule in the ASU (Figure 5), as also confirmed in the ^13^C CPMAS SSNMR spectrum. Also, for Form C, the crystal structure suggested a racemic nature, which was anticipated through polarimetric analysis, presenting an [α] of 0.0144 ± 0.0295 (*n* = 3).

Further, the FT-IR spectrum of Form C was very similar to that of Form B, highlighting a close relationship between these two polymorphs and a marked difference between the above and commercial Form A. The typical carbonyl bands at 1647 and 1624 cm^−1^ of the *syn* conformation of Form A are shifted in Form C and resonated at a closer frequency (1642 and 1631 cm^−1^), suggesting a conformation change in the *anti* PZQ coformer, as in the case of polymorph B [34]. Further characterization of Form C included solubility tests: the new solid showed a water solubility at 20 °C of 382.69 ± 9.26 mg/L, the highest value compared to Form B’s (281.31 ± 8.32 mg/L) and Form A’s (140.30 ± 9.26 mg/L), accordingly with the increasing melting point values of C, B, and A, respectively. Moreover, considering in vitro antischistosomal activity (against adult *Schistosoma mansoni*), Form C manifested an IC_50_ of 0.21464 μM after 72 h, comparable to that of Form A (0.1 μM) previously reported in the literature [97]. Finally, compared to previously found Form B, which was stable for more than one year, polymorph C was characterized by a dramatically lower physical stability (i.e., physically stable for 2 months), again in agreement with both its lowest melting point and highest water solubility among PZQ polymorphs. 

As for Form B, other subsequent works report alternative methods for the formation of Form C: specifically, Saikia et al. observed Form C (with a minute amount of Form A) via LAG with 5 μL of tetrahydrofuran (THF) [61], whereas Guedes Fernandes de Moraes et al. obtained Form C through (fast and slow) cooling crystallization in TEA both with low and high initial concentrations of PZQ Form A [62].

In 2021, Saikia and coworkers discovered two new polymorphic forms, namely Form D and Form E, via mechanochemistry by investigating different grinding techniques [61]. Specifically, Form D was obtained through NG of PZQ Form A for 5 h at 25 Hz. The melting point of this new form was detected at 133.3 °C through DSC analysis, and FT-IR spectroscopy confirmed the formation of Form D by evidencing some shifts: the C=O stretching frequency was observed at 1645 cm^−1^, which was shifted by about 2 cm^−1^ in comparison to Form A (1647 cm^−1^) and Form B (1641 cm^−1^); significant shifting was also observed in the aliphatic -CH stretching for polymorph D. 

LAG in the presence of 10 μL of THF or AcT gave, instead, a different outcome: DSC analysis showed a peak at 135.5 °C, thus indicating a possible new solid form designated as Form E; moreover, the new form presented a PXRD pattern clearly different from that of Form A, with the disappearance of the characteristic peak at 6.7° and the appearance of a very intense peak at 21.8°. SSNMR of Form E evidenced chemical shifts of the two carbonyl groups at 175.3/173.0 and 162.2 ppm, instead of 175.4/176.2 and 165.8/164.6/162.1 ppm, as reported for Form A.

Unfortunately, no suitable single crystals were grown to solve the crystal structures of the new Forms D and E by SXRD.

More recently, other two polymorphs of PZQ have been discovered and called Form G and H [62]. Starting from Form G, Guedes Fernandes de Moraes et al. obtained evidence of this new form by characterizing PZQ-DMA solvate through thermal analysis (the DSC curve showed a new endothermic event at 132.57 °C that was in between the desolvation event and the melting of Form A) and studying its physical stability by aging at ambient conditions: after 20 days, the PZQ-DMA solvate converted into a mixture of Form A and a new form, detected by previously unreported peaks at PXRD analysis. Pure Form G was obtained by the authors when PZQ-DMA was submitted to a water vapor-induced atmosphere overnight. As mentioned above, the new form showed an endothermic event at 132.57 °C attributable to its melting, as concomitant TGA experiments did not show any mass loss. Additionally, the new polymorphic form clearly exhibited a different habitus from the laths of PZQ-DMA, presenting smaller rod-like crystals (Figure 4). No further characterization has been conducted on this new form. 

The second polymorph, Form H, appeared by studying the effect of two process factors on the cooling crystallization of (RS)-PZQ in the presence of TEA. Precisely, cooling crystallization in TEA performed with filtration of the prepared solution before cooling and without agitation gave rise to a new metastable polymorphic Form H. A unique endothermic event at 79.59 °C, not corresponding to weight loss in TGA, was detected through DSC analysis and indicated as a melting event, thus proving Form H as the most unstable anhydrous polymorph of PZQ. This latter form exhibited small platy crystals through SEM images (Figure 4), but no more solid-state properties were reported.

## 5. PZQ Amorphous Forms

The literature provides little information about amorphous forms of PZQ. 

Several studies reported the possibility of amorphizing PZQ in the presence of a second compound through the formation of amorphous solid dispersions [17,29,30,31,32,33,34,36,37,50,51,56,57,98,99]. 

In 2018, Zanolla et al. reported the possibility of obtaining amorphous PZQ, without the presence of any second substance that enhances the amorphization, but through melt-quenching [52]. Specifically, an amorphous form of PZQ was obtained by scanning a sample at 10 K/min from 25 to 160 °C, cooling down to 0 °C at 20 K/min, and then re-heating to 100 °C at 20 K/min. The glass transition of amorphous PZQ was determined through modulated differential scanning calorimetry (MTDSC) and it was expressed as an inflection point in the reversible curve. The authors found PZQ glass transition at 37.70 °C, which was in good agreement with the theoretical value predicted through a published method [100].

In the same work, Zanolla and coworkers reported the possibility of obtaining amorphous PZQ with a certain residual crystallinity via NG at cryogenic temperatures using liquid nitrogen as cryogenic media. 

In a subsequent study in which Zanolla et al. were investigating mechanochemical process conditions leading to the formation of PZQ polymorph C, amorphous-nanocrystalline PZQ was observed when using lower mechanochemical energy (i.e., lower frequency and higher milling time) [60] (Figure 4). Worthy of notice is the fact that PZQ did not degrade upon milling when the drug was milled alone, contrary to what was observed in the case of the formation of amorphous dispersions, in which a diminished drug recovery was found and it resulted as highly dependent on the percentage and type of excipient used [33,50,52].

A later article showed a new procedure to amorphize PZQ starting from polymorph E and grinding for 1 h in the absence of solvent. The amorphous state was stable under ambient conditions for 2 weeks and it showed a recrystallization into polymorph B after a cycle of heating to 135 °C and re-cooling at room temperature [61]. 

In 2020, Salazar-Rojas et al. reported another method to obtain an amorphous form of PZQ like those presented by Costa and coworkers [30]: Form A was placed in a vacuum oven at 150 °C with 60 mmHg for 30 min; then, the molten drug was transferred in a desiccator to reach room temperature and stored below 30 °C. The confirmation of PZQ amorphization was given by optical microscopy (the solid did not show any crystalline habitus) and through classical solid-state characterization techniques: no characteristic melting point was detected in DSC, even though the authors did not report any glass transition value. Moreover, typical halo diffusion in PXRD and the broadening of signals through SSNMR emerged. Furthermore, the authors performed IDR, thus demonstrating a superior dissolution rate of 9.0 mg cm^−2^ h^−1^ compared to that of Form A (1.6 mg cm^−2^ h^−1^) [63].

In 2023, Guedes Fernandes de Moraes et al. also obtained amorphous PZQ by fast cooling crystallization of PZQ in the presence of MeOH/H_2_O mixtures with 50 and 50% *v*/*v* of H_2_O content [62].

## 6. PZQ Multicomponent Solid Forms

### 6.1. PZQ Hydrates

The study of hydrated solid forms is of great importance as water is often present during different preformulation studies and several stages of drug manufacturing. Water molecules contain both donor and acceptor hydrogen groups and show, therefore, the capability to form H-bonds with other compounds. One of the direct implications of hydrate formation is the modification of several physicochemical properties of the drug. Indeed, hydrated forms are known to be less soluble than the corresponding anhydrous drug, thus having an impact on its biopharmaceutical properties [101]. Based on that, a comprehensive study on the hydrated forms of a particular drug is often necessary. 

Contemplating the anthelmintic drug (RS)-PZQ, three different hydrates are reported in the literature.

First, PZQ hydrate was reported by Salazar-Rojas and coauthors, who obtained a monohydrate (PZQ-MH) as a peculiar pink solid: PZQ Form A was melted in a vacuum oven at 150 °C with 60 mmHg for 30 min; then, it was transferred to a humidity temperature-controlled chamber while being whisked and rapidly cooled with liquid nitrogen at 20 °C and 70% of relative humidity (RH %) [63]. The obtained solid was kept in a freezer (−20 °C) overnight and then stored below 30 °C in a closed flask for subsequent characterization.

Starting from the thermal analyses, the DSC curve of the new form exhibited two endothermic transitions: the first, as a large endothermic event—without any defined peak—starting at about 78 °C and ending at 130 °C, was attributed to the dehydration, and the second (144 °C) was assigned to the PZQ Form A melting point. TGA experiments showed a gradual weight loss in the large range of 80–120 °C (total of 5.4% *w*/*w*, agreeing with the theoretical value for a PZQ monohydrate). Additionally, the water content of PZQ-MH was evaluated through the official USP loss on drying test [102], at 105 °C up to constant weight (3 h), where the sample weight loss was of 94.6%, confirming the 1:1 stoichiometry. Sharp peaks clearly different from those of Form A were detected through PXRD, confirming the crystallinity of the sample. Passing to spectroscopic properties, PZQ-MH showed an intense peak at 3417 cm^−1^, confirming the presence of water in the crystal structure; in the region related to the carbonyl stretching vibration, the monohydrated form exhibited a single shifted carbonyl signal at 1626 cm^−1^, compared to the previously mentioned double peak at 1647 and 1624 cm^−1^ of anhydrous Form A, assuming that the carbonyl group is involved in the interaction with water, also visible from the deshielding of the carbonyl carbon atoms in SSNMR spectra. ^1^H NMR spectroscopy testified to the presence of water with an easily distinguished resonance at δ 1.63 ppm: the integral of the signal was compared to the doublets resonating in the 4.6–3.7 ppm region and used to check the 1:1 stoichiometry. 

Surprisingly, PZQ-MH showed a slightly higher dissolution rate (2.2 mg cm^−2^ h^−1^) than Form A (1.6 mg cm^−2^ h^−1^), contrary to the expectations for hydrates [101].

Despite all performed characterization analyses, no crystal structure of PZQ-MH was solved and deposited in the CSD.

In the same year of appearance of the above-described PZQ-MH, Zanolla and coworkers reported a novel PZQ racemic hemihydrate (PZQ-HH) obtained through mechanochemistry [64]. Several methods to achieve this hydrated form are reported in their work: (i) PZQ-HH could be produced starting from the commercial Form A through a two-step mechanochemical treatment (i.e., 30 min of NG and, subsequently, 1 h of LAG with H_2_O), passing through the formation of an amorphous intermediate; (ii) it could also be obtained in a one-step 1 h LAG process when using Form B as starting material; (iii) its formation was also observed via slurry experiments of PZQ polymorph B in three days (contrary to Form A, which never converted to PZQ-HH with the same technique).

The formation of PZQ-HH was also promoted by grinding PZQ Form A in the presence of seeds of preformed PZQ-HH (95:5 or 90:10 *w*/*w*) for 1 h of LAG with H_2_O: a few seeds of the hydrate were sufficient to enhance the complete transformation of Form A in PZQ-HH, giving the chance to Form A to convert into PZQ-HH in a one-step procedure, instead of the above-mentioned two-step mechanochemical treatment.

PZQ-HH is a white powder that consists of agglomerates of large plates with a porous aspect and a broad specific surface area (Figure 4).

The DSC curve of the novel solid presented a sharp dehydration endotherm at about 68 °C and two other endothermic events at 109.05 °C and 133.95 °C, attributable to the melting points of polymorph B and Form A, respectively. The same events were confirmed by hot-stage microscopy (HSM). Additionally, TGA was performed to evaluate the sample weight loss that was of 2.19%, corresponding to the theoretical value for a hemihydrate.

**Figure 6:**
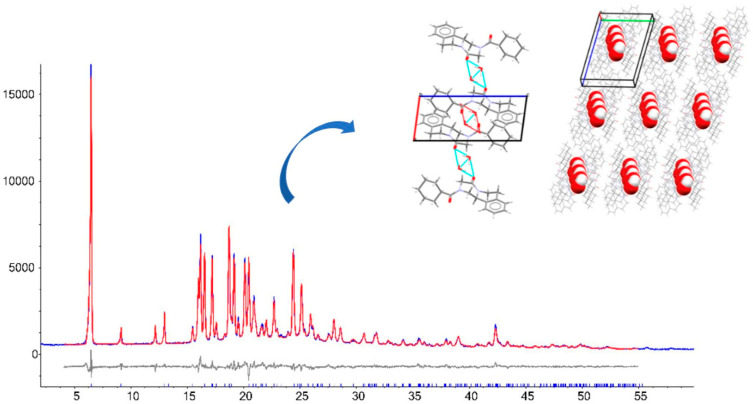
Rietveld refinement from the experimental pattern and crystal structure with packing and H-bonds motifs of PZQ-HH. Adapted from Reference [64].

The PXRD pattern of PZQ-HH was completely different from the already known PZQ solid forms: it presented characteristic sharp reflections that did not overlap either with those of Form A or with Form B and C and the enantiomeric hemihydrates. From the capillary PXRD pattern, the crystal structure of the new solid (indexed as WUHQAU in the CSD) was solved: PZQ-HH crystallizes in the triclinic space group of P-1 (as Form A), but just one independent molecule, rather than four as for TELCEU, is present in the ASU (Figure 6). In the crystal unit cell, every H_2_O molecule is linked to two PZQ entities (i.e., one (R)- and one (S)-) through H-bonds, confirming the 1:0.5 stoichiometry, and PZQ carbonyls exhibit the most common *anti*-conformation (similarly to B and C polymorphs and differently from Form A *syn* conformation). 

The ^13^C CPMAS SSNMR spectrum confirmed the formation of a new pure phase and the presence of only one molecule in the ASU, as attested by crystallographic data. SSNMR assignments for Cq indicated high similarities between PZQ-HH and PZQ Form B, while having significant differences from Form A.

The FT-IR spectrum of PZQ-HH presented a very sharp band at 3543 cm^−1^ due to the presence of H_2_O, differently from the anhydrous Form A. In the C=O stretching region, a single shifted broad band was detected at 1629 cm^−1^, instead of the typical doublet at 1647 and 1624 cm^−1^ of Form A, confirming the intermolecular interactions between the drug and water via PZQ carbonyl groups. 

Considering biopharmaceutical properties, PZQ-HH demonstrated improved water solubility (310.89 ± 3.07 mg/L) compared to Form A (217 ± 10.33 mg/L). Moreover, the IDR was of 0.0618 ± 0.0051 mg cm^−2^ h^−1^, like that attested for Form B (see Section 4) and twice that of the commercially available Form A (0.0299 ± 0.031 mg cm^−2^ h^−1^). As for the in vitro activity against adult *Schistosoma mansoni*, PZQ-HH exhibited an IC_50_ of 0.15 μM, identical to Form A (IC_50_ of 0.1 μM) [97].

The hemihydrated form demonstrated physical stability at ambient temperatures for 3 months; then, Form B started appearing. 

Interestingly, other authors noticed that PZQ-HH could also be obtained starting from PZQ monosolvate with acetic acid (PZQ-AA) by putting the latter in a water vapor atmosphere: in this condition, desolvation of AA and solvation of H_2_O occurred [62].

Considering these two above-discussed new hydrated forms, both PZQ-MH and PZQ-HH solid-state transformations under heat stress were monitored through hot-stage attenuated total reflectance coupled to mid infrared spectroscopy (HS-ATR-MIR) empowered by multivariate curve resolution coupled to alternating least squares (MCR-ALS) and DSC in a study reported by Salazar-Rojas et al. [103]. The monitoring setup involved heating the samples on the ATR stage at a rate of 5 °C/min (30–145 °C), while acquiring MIR spectra (3500–600 cm^−1^) every minute. MCR-ALS was used to resolve the high spectral overlapping evidenced among the species.

For PZQ-HH, it was previously established through DSC analysis that the dehydration initially generates polymorph B and, consequently, Form A appears [64]. Under HS-ATR thermal treatment, indeed, PZQ-HH totally converted into Form B at 90 °C; then, a second transformation occurred at 107 °C which quantitatively yields Form A at about 115 °C. Further heating gave rise to the last transition, observed at 136 °C, which corresponds to the complete melting of Form A. The MIR spectra, simultaneously acquired during the ATR stage, revealed a first change in the disappearance of the signal at 3485 cm^−1^ due to the loss of water during the transformation of PZQ-HH to Form B. Simultaneously, the splitting of ν(C=O) of the amide group (1634 cm^−1^) into two different vibrations (1643 and 1631 cm^−1^) and the displacement of one of them to a higher wavenumber were observed, showing the gain of rigidity in the covalent bonds and confirming the transformation into Form B. Subsequently, the conversion from Form B to Form A was displayed by the increasing of ν(C=O) of the amide groups, shifting from 1643 and 1631 cm^−1^ to 1645 and 1620 cm^−1^, respectively.

As for PZQ-MH, it is known that its dehydration undergoes a single-step transformation, directly converting into Form A [63]. Under the same above-mentioned HS-ATR thermal treatment, the MH dehydration process started approximately after 70 °C, which resulted in the concomitant formation of Form A and reached a maximum around 90 °C (90%). Then, the melting event of Form A occurred and became relevant after 125 °C. All the events detected were in full agreement with DSC observations. As in the case of PZQ-HH, the first change in the MIR spectra was the disappearance of the signal assigned to ν(OH) at 3481 cm^−1^, due to water loss during the conversion of PZQ-MH into Form A. Simultaneously, the ν(C=O) of the amide group (1624 cm^−1^) split in two vibrations with displacements to higher (1645 cm^−1^) and lower (1620 cm^−1^) wavenumbers, in accordance with the signals detected for Form A. 

In the same work, the authors also evaluated the effect of the thermal stress on PZQ-MH and PZQ-HH dissolution behavior. For this aim, the hemihydrate was heated at 84 and 124 °C for a period of 20 min (hereinafter referred as HH84 and HH124), whereas the monohydrate was kept at 124 °C for 20 min (referred as MH124). DSC and PXRD analyses of the three samples confirmed what was already clear from HS-ATR-MIR coupled to MCR-ALS: HH84 evidenced the presence of Form B with a small amount of amorphous (visible from the small baseline shift in PXRD); HH124 and MH124, instead, revealed the predominant presence of Form A, without traces of Form B and a little amount of amorphous. Considering the influence of this thermal stress on the dissolution of the solids, HH84 showed both an increase in the amount of drug dissolved at early times (10 min) and an increase in the dissolution rate in comparison to PZQ-HH. On the contrary, HH124 and MH124 presented similar behavior: early dissolution times due to the presence of the amorphous part, but a lower overall dissolution driven by the presence of Form A.

A later article reports another hydrated form found through supercritical CO_2_ processing. Specifically, MacEachern and coworkers observed the formation of the new phase using rapid expansion of supercritical solution (RESS) as a supercritical carbon dioxide (scCO_2_) processing method [65]. PZQ was wrapped in a Kimwipe and carefully placed in a 100 mL high-pressure vessel in an extractor oven (±0.5 °C). The vessel was pressurized with CO_2_ using an SFT-10 pump at 24 mL/min and sealed by closing the valves at the inlet and outlet. The vessel was held at 40 °C/17.9 MPa for 22.5 h and, after this period, purged with CO_2_ at the set pressure for 20 min at approximately 4–5 L/min. The pump was then stopped, and the vessel was de-pressurized. The solubilized solids were collected in the collection vials through a rapid expansion from the supercritical solution (RESS) process. The solid remaining in the Kimwipe after the experiment was simply exposed to high-pressure conditions for the duration of the experiment. 

Performing SEM analysis, the new form exhibited distinct needle-like morphology, with a larger particle size compared to that of anhydrous Form A (see Figure 4).

The novel form showed a complex thermal profile by DSC: the first endotherm with onset at 83.11 °C (peak 92.5 °C) was related to dehydration into a novel dehydrate (confirmed through variable-temperature PXRD); then, the novel dehydrate melted and recrystallized to Form A; the final endotherm occurred at 139 °C, agreeing with the melting of Form A. The TGA experiment detected a mass loss of 3.38% *w*/*w* in correspondence with the first endothermic event of DSC; also, KF titration attested to a water content of 3.24% *w*/*w*, in close agreement with the mass observed in TGA, confirming that the isolated solid was a hydrate with 0.6 equivalent (eq.) of water and, more precisely, a hemihydrate with 0.1 eq. excess water due to the hygroscopicity of the material.

The PXRD pattern of the new solid was indexed using PDXL2 software, and the solution with the best reliability was a monoclinic space group of P2/*m* with a unit cell volume of 2332.09 Å^3^ (a = 14.128 Å, b = 13.536 Å, c = 13.187 Å; α = γ = 90°, β = 112.368°) and a Z value of five for the unit cell. However, the scCO_2_ process did not generate single crystals for SXRD analysis; therefore, no crystal structure was solved and deposited in the CSD. 

In addition, IR analysis showed a band at 3586 cm^−1^, not visible in the case of anhydrous Form A and like the peak observed by Zanolla and coworkers (3543 cm^−1^), which was attributed to the C-OH signal typical of hydrates [64]. Moreover, some shifts of the C=O bands at 1624–1649 cm^−1^ were seen, like polymorphs B and C of PZQ, which were related to a change in the conformation of PZQ molecules from *syn* to *anti*.

Furthermore, the novel form demonstrated physical stability for at least 7.5 weeks under stressed conditions (40 °C and 75% RH) and exhibited pH-independent improved solubility in biorelevant media (FaSSGF pH 1.6 and FaSSIF pH 6.5) and water, showing 13–20% higher solubility compared to unprocessed PZQ Form A.

In a subsequent work, the same PZQ-HH was also found by fast cooling crystallization of PZQ in the presence of MeOH/H_2_O mixtures with 30 and 40% *v*/*v* of H_2_O and in EtOH/H_2_O mixtures with 40 and 50% *v*/*v* of H_2_O [62].

### 6.2. PZQ Solvates

It is well known from the literature that solvates are usually discovered either by chance during a specific manufacturing process or via systematic polymorph screening programs such as solution crystallization, slurry, and, more recently, mechanochemistry [104].

Few PZQ solvates have been discovered so far and they were found through cooling crystallization and mechanochemical methods.

The first two PZQ solvates were discovered through a mechanochemical screening performed with several liquids [66]. Specifically, 2-pyrrolidone (2-pyr) and acetic acid (AA) were found to be suitable liquids to give two PZQ solvates (PZQ-2P and PZQ-AA) with a 1:1 stoichiometry by grinding the drug in the presence of each solvent, regardless of the quantity added (η values tested in the range 0.05–0.5 μL [105]), for 30 min at 25 Hz. The same outcome was observed when starting from anhydrous polymorph B of PZQ.

Moreover, PZQ-2P and PZQ-AA could also be obtained by suspending an excess of PZQ Form A in 2-pyr and AA, thus performing slurry experiments. 

SEM images of PZQ-AA showed layered blocks with flat particles, with a rough surface presenting some holes, while PZQ-2P exhibited flat agglomerates with very thin needles on the surface (see Figure 4).

**Figure 7:**
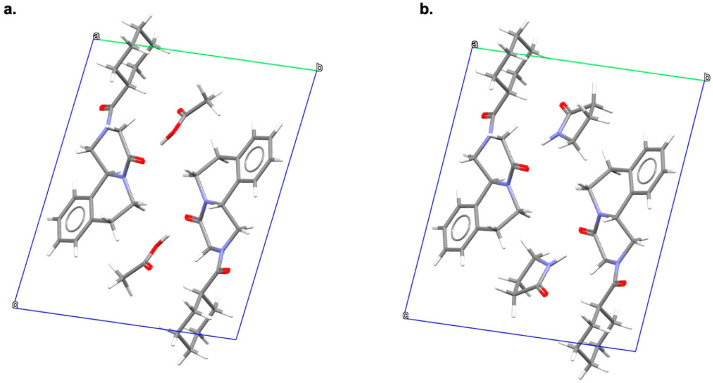
Crystal structures of (**a**) PZQ-AA and (**b**) PZQ-2-pyr solvates.

PXRD patterns of the two solvates showed reflections clearly different from PZQ Form A and other known PZQ solid forms, being, instead, quite similar and suggesting isostructurality between the two new forms. Synchrotron X-ray diffraction, through which their crystal structures were solved, confirmed that the solvates are isostructural: they both crystallize in the triclinic space group of P-1, showing one PZQ molecule linked via a strong H-bond to one molecule of solvent in the ASU (Figure 7). Intermolecular interactions and stoichiometry were confirmed through FT-IR and SSNMR analyses.

Considering FT-IR results, PZQ-2P and PZQ-AA displayed the stretching of the heterocyclic carbonyl (originally at 1647 cm^−1^ in Form A) at 1638 cm^−1^, thus showing a lower frequency difference in the carbonyl vibrations than that observed for Form A and confirming the presence of an *anti*-conformation. Moreover, the ν(N-H) band, originally present in 2-pyr at 3488–3474 cm^−1^ and not present in pristine PZQ, resulted in a downward shift in the solvate (3243 cm^−1^), confirming both the presence of 2-pyr in the structure and the intermolecular interaction between the two coformers. As for PZQ-AA, two bands detected at 2600 cm^−1^ were helpful in confirming the presence of AA in the structure of the solvate.

Passing to the ^13^C CPMAS SSNMR spectra of PZQ-2P and PZQ-AA, the presence of the solvent molecules was easily detected by peaks at 172.1 (COOH) and 19.7 (CH_3_) ppm for PZQ-AA and at 177.5 (C=O) and 19.4, 28.9, and 40.4 (CH_2_) ppm for PZQ-2P. Additionally, in the case of PZQ-AA, one of the carbonyl signals was not visible due to the overlapping with the COOH signal of AA (172.1 ppm), whereas it was recognizable in PZQ-2P. In both spectra, the number of signals was consistent with the 1:1 stoichiometry.

PZQ-2P and PZQ-AA were indexed in the CSD as DAJCAW and DAJCEA, respectively.

Considering their thermal behavior, PZQ-2P and PZQ-AA were characterized by a single endothermic event, imputable to desolvation, at 85.69 °C and 72.26 °C, respectively.

In terms of water solubility and IDR analyses, the two solvates showed a superior biopharmaceutical performance (roughly double IDR) when compared to the anhydrous (RS)-PZQ.

Interestingly, kept at room temperature in sealed vials, PZQ-2P and PZQ-AA provided a physical stability of at least 18 months. Additionally, mechanical treatment at 25 Hz for 200 min was ineffective on the stability of the two monosolvated forms.

Subsequently, PZQ-AA was also obtained through fast and slow cooling crystallization both with low and high initial concentrations of (RS)-PZQ [62].

Later, Guedes Fernandes de Moraes et al. reported the discovery of a dimethylacetamide (DMA) solvate of PZQ [62]. Specifically, PZQ-DMA solvate was obtained through fast cooling crystallization of PZQ Form A: two different levels for the initial concentration of PZQ (low and high) and two for the cooling rate [slow (0.2 °C/min) and fast (5 °C/min)] were investigated and PZQ-DMA solvate was obtained with low and high concentrations of PZQ but just with rapid cooling crystallization, whereas the slow cooling method gave the same starting Form A. The authors observed a completely different PXRD pattern from that of TELCEU or other reported PZQ solid forms, and thermal analyses were essential to prove that the new form was a solvate. The DSC curve showed an endothermic event attributable to desolvation at 58 °C, followed by two melting events corresponding to the melting of Form G and the melting of Form A, respectively (see Section 2.1 and Section 4). The desolvation was also confirmed through the TGA experiment by a weight loss of approximately 20% *w*/*w*, agreeing with the theoretical value for a monosolvate. Moreover, SEM images demonstrated the presence of lath-shaped crystals for the new solvate, differing from the smaller acicular crystals of Form A (Figure 4).

Even though the formation of a new phase was clear from all the solid-state characterization analyses, no PZQ-DMA crystal structure was solved and deposited in the CSD.

### 6.3. PZQ Cocrystals

Cocrystallization represents a growing strategy to obtain new crystal forms in the context of pharmaceutical science [106]. Indeed, pharmaceutical cocrystals, formed by an active pharmaceutical ingredient (API) and one or more cocrystal formers, are highly interesting due to their effect on physicochemical properties (i.e., solubility, bioavailability, mechanical/humidity/thermal stability, and compressibility) and their role in separation technologies, particularly for chiral molecules [107,108,109,110,111,112,113,114,115].

Considering that the molecular structure of PZQ does not contain salt-forming functional groups (but just two carbonyl groups acting as H-bond acceptors) and the possibility to create diastereomeric cocrystal pairs for its chiral resolution, PZQ’s propensity for cocrystal formation has recently attracted great attention.

Espinosa-Lara and coworkers explored for the first time PZQ’s cocrystallization tendency by combining (RS)-PZQ and aliphatic dicarboxylic acids via LAG, assuming that cocrystallization might be induced by the formation of dimeric heterosynthons [58]. Specifically, PZQ was combined with oxalic acid (OXA), malonic acid (MALO), succinic acid (SUC), maleic acid (MALE), fumaric acid (FUM), glutaric acid (GLU), adipic acid (ADI), pimelic acid (PIM), suberic acid (SUB), azelaic acid (AZE), and sebacic acid (SEB) in a 2:1, 1:1, and 1:2 molar ratios in two different solvents (i.e., AcT and ACN). Only SUB, AZE, and SEB did not allow PZQ cocrystallization and nine new cocrystals were discovered. Comparison of the PXRD patterns indicated that OXA, MALO, SUC, MALE, FUM, and GLU generated a 1:1 cocrystal (i.e., PZQ-OXA, PZQ-MALO, α-PZQ-SUC, PZQ-MALE, PZQ-FUM, and PZQ-GLU), while ADI and PIM gave a 2:1 cocrystal (PZQ-ADI and PZQ-PIM). Also, all FT-IR spectra did not correspond to a sum of the starting materials and significant shifts were seen in PZQ carbonyl vibration bands, reflecting the various H-bonding patterns of the cocrystals. The C=O stretching vibrations of the starting coformers ranged from 1658 to 1704 cm^−1^ and were shifted to larger wavenumbers in the cocrystals, requiring higher energy for the stretching, which is consistent with the formation of weaker interactions (i.e., C-H···O and O-H···O).

**Figure 8:**
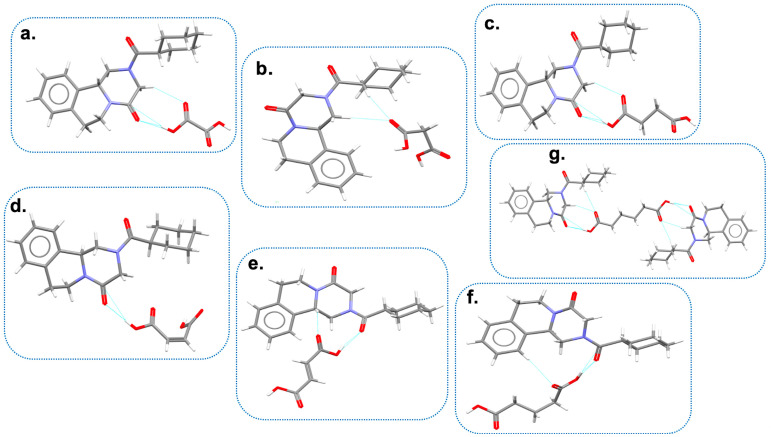
H-bonds motifs and ASUs of first seven cocrystals discovered: (**a**) PZQ-OXA; (**b**) PZQ-MALO; (**c**) β-PZQ-SUC; (**d**) PZQ-MALE; (**e**) PZQ-FUM; (**f**); PZQ-GLU; (**g**) PZQ-ADI.

To solve the crystal structure of the new solids, PZQ and each coformer were also dissolved in 2:1, 1:1, 1:2, 1:3, and 1:4 molar ratios in hot AcT or ACN in the presence of a small quantity of mechanochemically preformed cocrystals as seeds to perform classical solution crystallization. This procedure allowed the isolation of single crystals suitable for SXRD analysis in the case of PZQ-OXA, PZQ-MALO, PZQ-MALE, PZQ-FUM, PZQ-GLU, and PZQ-ADI. PZQ-PIM, instead, did not give single crystals and, therefore, its crystal structure was not solved. In case of using PZQ and SUC as coformers, solution crystallization provided single crystals showing different PXRD patterns from that observed with α-PZQ-SUC, thus giving rise to a polymorphic form, namely β-PZQ-SUC (Figure 8), as also attested to from the similarity of the two FT-IR spectra. 

PZQ-OXA, β-PZQ-SUC, and PZQ-GLU (indexed in the CSD as TELCOE, TELDAR, and TELDIZ, respectively) crystallized in the same space group (P-1) and had similar unit cell edge lengths, suggesting similarities in the supramolecular organization of the three cocrystals: each of the COOH group of the acids was connected to a PZQ molecule through double or triple bridged heterodimeric motifs containing O-H···O H-bonds and C-H···O contacts. The ASU of the three cocrystals comprises one molecule of PZQ with carbonyls in the *anti*-conformation and one molecule of each coformer, confirming the 1:1 stoichiometry already established by PXRD. In PZQ-SUC and PZQ-GLU, PZQ molecules were bound together through double-bridged homodimeric motifs. 

PZQ-FUM (refcode TELBUJ in the CSD) crystallizes in the space group of P-1 and shows analogous connectivity of PZQ-OXA. An important difference was that, in the case of PZQ-FUM, there were two sets of independent molecules in the ASU. 

PZQ-MALE (TELCIY in the CSD), instead, crystallizes in the orthorhombic space group Pna2_1_ and shows a connectivity like PZQ-SUC, with additional C-H···O contacts between MALE molecules. Both PZQ-FUM and PZQ-MALE displayed an *anti*-conformation of carbonyls of PZQ molecules.

Somewhat different was the case of PZQ-MALO (indexed as TELDEV in the CSD): this cocrystal crystallized in the P2_1_/*c* space group and was the unique new system which presented the same *syn* molecular conformation of PZQ Form A. This configuration allowed for the formation of 26-membered cyclic [2 + 2] aggregates: the variability in the connectivity occurred because MALO entities were disordered over two positions, giving rise to [2 + 2] assemblies with slightly different conformations. Additionally, MALE molecules were bound together to further stabilize the crystal structure.

PZQ-ADI (refcode TELCAQ in the CSD) crystallizes in the P-1 space group with a 2:1 stoichiometry; therefore, the larger quantity of PZQ favored the formation of homodimeric motifs (PZQ-PZQ entities) to form double chains linked to the coformer. In case of PZQ-ADI, a low temperature crystal structure was also indexed (refcode TELCAQ01), which confirmed that the crystal structure was quite compact.

To summarize, in all cocrystalline structures, the dominant H-bonding interactions consisted of heterodimeric motifs formed between PZQ and dicarboxylic acids. Seven of the nine motifs were characterized by O-H···O H-bonds and occurred with either of the C=O groups of PZQ. Additionally, PZQ molecules were bound together through homodimeric H-bonds and there was only one case showing double-bridged H-bonding contacts between molecules of the cocrystal former, that is, PZQ-MALO. Worthy of notice is that the *anti*-conformation was adopted for all new cocrystals, except for PZQ-MALO, which exhibited the same *syn* conformation of (RS)-PZQ Form A.

Physicochemical properties (i.e., thermal and spectroscopic behavior, solubility, dissolution), oral bioavailability, and pharmacokinetic parameters of some previously discussed cocrystals were deeply investigated by Wasim et al. [116]. Precisely, their aim was to elucidate the relationship between the physicochemical properties and pharmacokinetic parameters (i.e., Cmax, Tmax, and area under the curve, AUC) of PZQ cocrystals and the chain lengths (=n) of the selected dicarboxylic acid coformers, namely OXA (n = 0), MALO (n = 1), SUC (n = 2), GLU (n = 3), and ADI (n = 4). In the same work, they also assessed the effect of a polymer (hydroxy propyl cellulose, HPC) on PZQ cocrystal formation. 

Firstly, cocrystals were prepared using the slurry crystallization method, and the results were evaluated by comparing their PXRD patterns with the respective PXRD patterns reported in the CSD.

Concerning FT-IR analyses, all the spectra of PZQ cocrystals were different from the starting materials. The two carbonyl amidic groups of pure PZQ were detected at 1645 and 1622 cm^−1^, being in accordance with previously reported values. In the case of PZQ cocrystals, carbonyl stretching ranged from 1593 to 1630 cm^−1^, and this downward shifting justified changes in H-bonding patterns in the resulting cocrystals. The C=O stretching vibrations of dicarboxylic acid coformers ranged from 1664 to 1697 cm^−1^, while in the cocrystals these vibrations ranged from 1712 to 1742 cm^−1^: the shifting of carbonyl stretching vibration to a larger wavenumber requires further energy, which is consistent with weaker intermolecular interactions (C-H···O and O-H···O) [58]. According to the thermal behavior, DSC and TGA analyses were both performed. No dehydration was seen in all cocrystals and cocrystal melting points were different from the starting materials. Precisely, melting points were detected at 159.9, 147.7, 141.2, 126.2, and 122.9 °C for PZQ-OXA, PZQ-MALO, PZQ-SUC, PZQ-GLU, and PZQ-ADI, respectively, showing a consistent effect between the spacer group of each coformer and the cocrystal melting point: the higher the number of carbons in the coformer space group, the lower the cocrystal melting event. 

From the TGA results, it was evident that PZQ-OXA, PZQ-MALO, and PZQ-SUC started to dissociate at about 163, 147, and 167 °C, respectively, whereas PZQ-GLU and PZQ-ADI were thermally more stable and started to decay at 199 and 208 °C, respectively. 

Passing to biopharmaceutics aspects, results from the solubility studies showed a substantial improvement of PZQ cocrystal aqueous solubility when compared to pure PZQ. Indeed, pure PZQ showed a solubility of 0.392 ± 0.1 mg/mL, in agreement with literature data [13,76], while the highest and lowest PZQ cocrystal solubilities observed were 10.5 ± 1.7 and 3.7 ± 1.08 mg/mL, respectively. 

All cocrystals showed an improved dissolution profile with a descending order at 90 min as PZQ-SUC (68%), PZQ-ADI (57.96%), PZQ-GLU (54.05%), PZQ-OXA (51%), and PZQ-MALO (46.05%). 

According to the pharmacokinetics results, an enhanced oral bioavailability in rabbits was observed for all cocrystals compared to pure PZQ (AUC 10.06 ± 2.51 μg h/mL, with PZQ-SUC exhibiting the highest oral bioavailability (33.84 ± 6.05 μg h/mL) and PZQ-MALO the lowest (15.40 ± 3.65 μg h/mL). Furthermore, the cocrystals Cmax reached in 1 h were higher compared to pristine PZQ, except for PZQ-OXA and PZQ-MALO. 

The results of solubility, in vitro dissolution tests, and oral bioavailability were all consistent, as the enhanced solubility was translated into an improved dissolution and oral bioavailability. On the contrary, no consistency of the spacer group effect was seen in the biopharmaceutical properties and pharmacokinetics parameters. 

Finally, PZQ cocrystals were also prepared via slurry in the presence of HPC to investigate the effect of polymer on their preparation. The results showed that HPC did not inhibit PZQ cocrystal formation except for PZQ-SUC, as PXRD analysis evidenced prominent peaks of residual PZQ in that sample.

Recently, Salas-Zúñiga and coauthors combined the potential of the previously obtained PZQ cocrystals with size reduction by confinement in mesoporous silica material (i.e., SB-15) with nanopores (ca. 5.6 nm pore size) to further improve the solubility and dissolution of the materials [98]. Among the seven previously solved new cocrystals, PZQ-GLU was chosen for the following reasons: (i) it had a smaller unit cell volume than racemic PZQ; (ii) it melted at a lower temperature than raw (RS)-PZQ (ΔT = −15.8 °C), allowing it to perform the melt loading method for confinement; (iii) its recrystallization from the melt gave only the original cocrystal and displayed a unique fingerprint in the IR spectrum, thus making it easily recognizable. Four SBA-15/cocrystal *w*/*w* ratios were prepared, and, for comparison, the same inclusion experiments were carried out with pristine PZQ in the same silica material. N_2_ adsorption–desorption analysis, PXRD, IR spectroscopy, ^13^C SSNMR, DSC, and field-emission SEM (FE-SEM) were used to characterize the nanoconfined materials and compared to SB-15, raw PZQ, and pure PZQ-GLU cocrystal. N_2_ adsorption–desorption analysis showed a complete filling of the available channels of Sb-15 for the composition 50:50 *w*/*w* and, therefore, the composites with 50:50 *w*/*w* ratio of SBA-15/PZQ and SBA-15/PZQ-GLU were selected for further studies. FE-SEM images evidenced a rod-like morphology for SBA-15/PZQ-GLU instead of the smooth surface appearance of the micrometer-sized PZQ-GLU crystals. The PXRD pattern of SB-15/PZQ-GLU presented a broad halo, characteristic of raw amorphous SBA-15, with low-intensity diffraction peaks overlapping with the PZQ-GLU cocrystal, while SBA-15/PZQ exhibited the typical halo diffusion for amorphous material, and no peaks of crystalline PZQ were seen. As for the FT-IR results, the two C=O stretching vibrations of the GLU coformer at 1612 and 1715 cm^−1^ also remained in SBA-15/PZQ-GLU, supporting the cocrystal presence in the nanoconfined compound. For SBA-15/PZQ, the two C=O bands of raw PZQ (1647 and 1624 cm^−1^) were joined as one broad band at 1645 cm^−1^, behavior already observed by Perissutti and coworkers in amorphous dispersions [50]. Further, a comparison of ^13^C CPMAS and SPE (Single Pulse Excitation) allowed the conclusion that SBA-15/PZQ-GLU was a mixture of a cocrystalline bulk solid (outside the mesopores) and a more mobile phase (inside the mesopores), the latter being more evident in composites at lower ratios of PZQ-GLU. Through thermal analysis, SBA-15/PZQ did not show a distinct melting transition, confirming the amorphous state of the nanoconfined drug; SBA-15/PZQ-GLU presented two broad endothermic transitions at 63.0 and 117.2 °C, confirming the copresence of two types of phases: the cocrystalline bulk solid outside the mesopores was detected at 117.2 °C, like pure PZQ-GLU melting at 123.0 °C, and the more mobile phase inside the mesopores at 63.0 °C, as nanoconfined drugs are known to display a huge depression of their melting temperature [117,118,119]. To summarize, several characterization techniques demonstrated that racemic PZQ was loaded mainly in amorphous form, whereas a more mobile/solid-like phase of PZQ-GLU was nanoconfined within SBA-15. The contrasting behavior of the two nanoconfined solids was attributed to the more robust O-H_GLU_···O=C_PZQ_ intermolecular H-bonds in the cocrystal in comparison to C-H_PZQ_···O=C_PZQ_ contacts in (RS)-PZQ and to a larger crystal lattice volume of PZQ over the PZQ-GLU cocrystal (3320.1 vs. 1167.9 Å^3^).

The impact of pure PZQ and the cocrystal confined in SBA-15 was further examined by performing dissolution studies in two different experimental settings: (i) under non-sink conditions with a saturation index (SI) [120] of 0.014 for PZQ and 0.02 for PZQ-GLU, in the presence of a precipitation inhibitor polymer (Methocel 60 HG), and (ii) under non-sink conditions with an SI of 1.99 for PZQ and PZQ-GLU in aqueous HCl pH 1.2 at 37 °C. In the first case, SBA-15/PZQ-GLU showed a sustained solubilization of PZQ, increasing the AUC_0–90min_ up to 5.1-fold in comparison to pristine PZQ, similarly to SBA-15/PZQ. Under the second condition, the nanoconfined composite generated an immediate release of the drug. Thus, this study confirmed that nanoconfinement combined with cocrystallization is an efficient tool to further improve some properties of poorly soluble drugs like PZQ.

Finally, focusing the attention, once again, on PZQ-GLU plus also PZQ-SUC cocrystals, the same scientific group reported two novel cocrystals obtained through LAG in the presence of the above-mentioned coformers ground with enantiomerically pure (R)-PZQ in a 1:1 molar ratio [67]. The new cocrystals (i.e., (R)-PZQ-GLU and (R)-PZQ-SUC) were deeply characterized and compared with previously reported analogous cocrystals obtained with racemic PZQ (hereinafter referred as (RS)-PZQ-GLU and (RS)-PZQ-SUC), pure (R)-enantiomer hemihydrate (i.e., (R)-PZQ-HH), and pristine (RS)-PZQ. PXRD patterns of the new phases clearly differed from both starting materials and (RS)-PZQ-GLU and (RS)-PZQ-SUC. Suitable single crystals for SXRD analysis were obtained through classical solution crystallization and their simulated PXRD patterns agreed with experimental ones. (R)-PZQ-GLU and (R)-PZQ-SUC (indexed as KEQVEL and KEQVAH in the CSD, respectively) crystallize in a chiral space group (P2_1_), confirming the enantiomerically pure nature of PZQ. Structural analysis revealed that the dominant supramolecular interactions in the new crystals were quite similar: both amide groups of (R)-PZQ were involved in heterosynthons consisting of O-H_coformer_···O=C_PZQ_ H-bonds with the COOH groups of the dicarboxylic acid. Also, complementary secondary (N)C-H_PZQ_···O=C_coformer_ interactions generated two slightly distinct eight- and nine-membered cyclic double bridged heterodimeric synthons. 

In the IR spectra of (R)-PZQ-GLU and (R)-PZQ-SUC, the band observed at roughly 3500 cm^−1^ for the H_2_O molecules in starting (R)-PZQ-HH was missing, highlighting the anhydrous nature of the two cocrystals. Moreover, C=O stretching vibrations of PZQ were displaced at smaller wavenumbers, while C=O bands of coformers were displaced at larger wavenumbers: these shifts suggested that H-bond interactions in the cocrystals were stronger than those observed between H_2_O molecules and (R)-PZQ in the hemihydrate and those of dicarboxylic acid dimers. 

The thermal behavior of the two cocrystals was slightly different: (R)-PZQ-SUC presented a melting point at 130.2 °C, which was in between the starting materials (112.9 °C for (R)-PZQ-HH and 184.9 °C for SUC), whereas (R)-PZQ-GLU exhibited a melting temperature of 81.0 °C, being lower than the individual components (112.9 °C for (R)-PZQ-HH and 102.9 °C for GLU). This behavior could find a possible explanation in the difference of stability, solubility, and the dissolution rate of the two systems. Therefore, the authors performed IDR and powder dissolution studies on the two enantiomerically pure cocrystals and compared them to the corresponding racemic (RS)-PZQ-GLU and (RS)-PZQ-SUC, pristine (R)-PZQ-HH, and (RS)-PZQ in a simulated gastric dissolution medium (HCl, pH 1.2, 37 °C) where dicarboxylic acids were fully protonated. IDR results showed that all four cocrystals (both enantiomerically pure and racemic) had superior dissolution compared to (RS)-PZQ and (R)-PZQ-HH, with advantages by factors ranging from two to five, depending on the cocrystal, with respect to (R)-PZQ-HH. The IDRs were in the order (R)-PZQ-GLU > (RS)-PZQ-SUC ≈ (RS)-PZQ-GLU > (R)-PZQ-SUC. Interestingly, the IDRs of the four cocrystals exhibited a slope that changed significantly in the range of 30–60 min, which was attributed to a phase transformation into (R)-PZQ-HH and (RS)-PZQ-HH, as attested to by IR and PXRD analyses of the IDR tablets. In powder dissolution tests, these studies were performed in non-sink conditions by adding dissolution medium, simulating the gastric fluid to an excess of cocrystal, to assess the supersaturation potential of the enantiomerically pure cocrystals and their racemic counterparts. In this case, (R)-PZQ-GLU, (RS)-PZQ-GLU, and (RS)-PZQ-SUC showed increased solubility compared to (RS)-PZQ and (R)-PZQ-HH, giving maximum concentrations of 4.6 mM, 3.8 mM, and 2.7 mM, respectively, within the first 5 min. On the contrary, for the enantiomerically pure (R)-PZQ-SUC cocrystal, the dissolution profile was like that of (R)-PZQ-HH, suggesting no generation of a supersaturated solution. 

After a period of approximately 5 min, the concentration of the three cocrystals decayed to a concentration like (RS)-PZQ and (R)-PZQ-HH, indicating a rapid reprecipitation. PXRD analysis on the recovered samples suggested a conversion of the cocrystals to the parent compound (RS)-PZQ and (R)-PZQ-HH, as in the case of IDR.

Coming back to the cocrystallization strategy, three years after the discovery of PZQ cocrystals with dicarboxylic acids [58], Sánchez-Guadarrama et al. used cocrystallization to achieve a chiral resolution of racemic PZQ via the formation of cocrystal diastereoisomers with L-Malic Acid (L-MAL), as discussed in detail in paragraph 3. In short, the authors performed LAG with 10 μL of AcT using a 1:1 stoichiometric mixture of (RS)-PZQ and L-MAL for 30 min at 25 Hz and obtained two enantiomerically pure cocrystals (i.e., (R)-PZQ/L-MAL and (S)-PZQ/L-MAL), the structures of which were solved and deposited in the CSD [68].

Similarly, Cugovčan and coworkers reported having obtained PZQ cocrystals with citric (CA), malic (MAL), salicylic (SA), and tartaric acids (TA) by means of LAG [39]. Precisely, (RS)-PZQ was milled in the presence of each coformer in a 1:1 stoichiometry for 30 min at 25 Hz with or without an addition of 15 μL of absolute EtOH, LAG being the most efficient technique. PXRD patterns of PZQ-CA, PZQ-MAL, PZQ-TA, and PZQ-SA showed several new peaks and no residual peaks of the starting materials, confirming the cocrystal formation in a 1:1 molar ratio. DSC analyses revealed a new endothermic peak with an onset of 51.07 °C for PZQ-CA and 150.81 °C for PZQ-TA, whereas DSC was not useful to elucidate melting events in the cases of PZQ-MAL and PZQ-SA. Further information about the cocrystal formation was, instead, obtained by FT-IR analysis. The two typical carbonyl stretching vibrations of PZQ Form A observed at 1647 and 1624 cm^−1^ were downward shifted and only one broader band was noticed at 1612, 1593, and 1606 cm^−1^ for PZQ-MAL, PZQ-SA, and PZQ-TA, respectively, suggesting H-bond interactions between PZQ and cocrystal formers and an involvement of both carbonyl groups in H-bond formation. Additionally, the shift of band from 1653 to 1670 cm^−1^, assigned to C=O stretching vibrations of the carboxylic group of SA, confirmed the formation of the H-bond between PZQ carbonyl groups and the SA carboxylic group. Passing to MAL, considerable differences in band intensities at 3438 and 1683 cm^−1^ characteristic for -OH and C=O of the coformer could be noticed, indicating that the hydroxyl group of MAL probably participated in the H-bonds with PZQ carbonyl groups. In the case of TA, bands at 3402 and 3330 cm^−1^, assigned to the stretching of the alcoholic group of TA, resulted in a shift in PZQ-TA, and a new band around 3480 cm^−1^ also emerged, confirming the interaction with PZQ carbonyl groups through the TA alcoholic group. Worthy of notice is the case of PZQ-CA, in which for the FT-IR spectrum no shifts of characteristic bands of PZQ were detected, while a shift of the C=O group of the coformer was noticed from 1751, 1745, and 1699 cm^−1^ to 1753, 1726, and 1691 cm^−1^ that anyway suggested the formation of a new solid form. All the prepared cocrystals showed pH-dependent solubility, with the highest saturation solubility observed at pH 4.5, since the ionizable groups of coformers were all ionized at that value, as evident from their pKa [121,122]. Among the four cocrystals, the PZQ-MAL cocrystal showed the highest solubility. Considering the dissolution studies, all of the new systems presented significantly superior dissolution properties compared to those of the pure drug. Interestingly, the authors noticed that PZQ-MAL was chemically unstable and favored PZQ photodegradation. Despite these characterizations, no new crystal structure was solved and reported in the CSD.

Subsequently, Yang et al. published a work in which conformations of PZQ and three flavonols used as coformers, namely kaempferol (KAE), quercetin (QUE), and myricetin (MYR), were analyzed through theoretical calculations to predict cocrystal formation [69]. They based their study on molecular electrostatic potential surfaces (MEPS) using density functional theory (DFT), which can accurately reflect changes in the intermolecular interaction sites caused by the conformational changes. This system provides a useful method for predicting the interactions of the participant molecules in a cocrystal [123]. Precisely, they started from the determination of the conformations of flavonols and PZQ Form A by means of DFT and their Boltzmann distributions at 300 K. Then, MEPS for each conformation were calculated to predict the difference in the interaction site pairing energies (ΔE) and, therefore, the cocrystal formation. As is well known from the literature, the smaller the ΔE value, the greater the probability of forming cocrystals [123].

KAE, QUE, and MYR exhibited two main conformations. For KAE and MYR, the difference among them was very small; for QUE, the two conformations differed more greatly due to the asymmetric distribution of the hydroxyl groups: according to the CCDC, conformation 1 (QUE C1) accounts for approximately 75% and conformation 2 (QUE C2) for roughly 25%. PZQ showed four main conformations due to the rotation of the cyclohexylcarbonyl, showing two of them with the carbonyl groups on the opposite site (*anti* conformation) (i.e., C1 and C2) and the other two with the carbonyl groups on the same site (*syn* conformation) (i.e., C3 and C4). The CCDC reports a predominancy of conformations C1 and C3 for PZQ. ΔE values, calculated considering the effective number of H-bond acceptors and donors of PZQ and flavonols, enabled the prediction of four different cocrystals, namely PZQ-KAE, PZQ-QUE 1, PZQ-QUE 2, and PZQ-MYR. For KAE, the two maxima of the MEPS were located on the region of the hydroxyl groups which could form H-bonds, with the two minima of MEPS on PZQ C1. Considering that the two hydroxyl sites were located on both sides of the KAE molecule, the cocrystal was speculated to be composed of a stoichiometric ratio of 2:1 PZQ-KAE. For QUE C1, there were two pairs of maxima of the MEPS located on the hydroxyl groups that could interact with the two minima of MEPS on PZQ. For QUE C2, the two maxima sites of MEPS located in the region of the hydroxyl groups could only interact with the minima of MEPS on PZQ C3. For MYR, the situation was the same observed for QUE C2: the interaction was supposed to be only with PZQ in conformation C3. 

**Figure 9:**
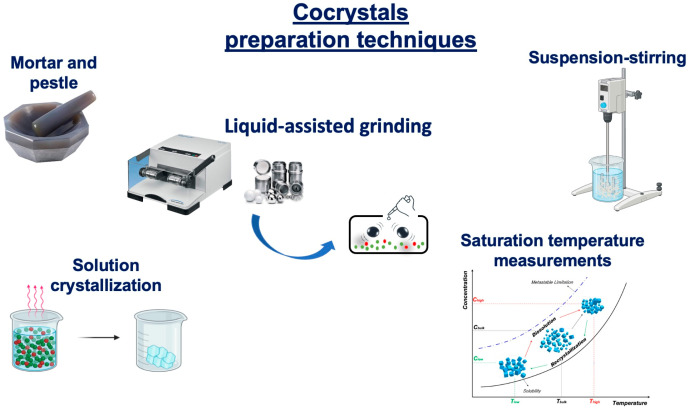
Main preparation techniques used for PZQ cocrystals. Adapted from references [69,70,71,73] and https://app.biorender.com (accessed on 14 December 2023).

All four predicted cocrystals were obtained experimentally by means of the suspension-stirring method (Figure 9) and characterized through PXRD. For KAE, the PZQ-KAE cocrystal was obtained in a 2:1 stoichiometry. For QUE, two kinds of cocrystals were achieved as assessed by PXRD. The stoichiometric ratio of one was 2:1 and the single crystal, named PZQ-QUE 1, was also grown. The stoichiometric ratio of the other cocrystal, PZQ-QUE 2, was 1:1, but no single crystal was obtained in that case. For MYR, the cocrystal PZQ-MYR showed a 1:1 stoichiometric ratio and the single crystal was also grown. 

The three single crystals were all block-shaped crystals and their structural analysis by SXRD clarified the type of interactions of the molecules in the cocrystal packing. PZQ-KAE (indexed as ANAYOG) crystallizes in the monoclinic space group P2_1_/*a* and has four formula units per unit cell (Z = 4). The asymmetric unit contains one KAE and two PZQ molecules, consistent with theoretical calculations, with the presence of PZQ conformation C1 (*anti*-conformation). PZQ-QUE 1 (ANAYUM in the CSD) crystallizes in the triclinic space group P-1 and shows two formula units per unit cell (Z = 2). The asymmetric unit contains one QUE and two PZQ molecules with both PZQ conformations C1 and C3, thus presenting one molecule in *anti* and the other in *syn,* both interacting with QUE. PZQ-MYR (ANAZAT in the CSD) crystallizes in the monoclinic space group P2_1_/*c* and has four formula units per unit cell (Z = 4). The asymmetric unit contains one MYR and one PZQ molecule, consistent with the 1:1 stoichiometry, with the presence of PZQ conformation C3 (*syn* conformation). Therefore, the SXRD results were demonstrated to be consistent with the predicted interactions. 

Experimentally obtained cocrystals were also characterized through DSC and FT-IR spectroscopy. All four cocrystals presented a single melting peak which was in between the starting materials, with increasing values in the order PZQ-KAE < PZQ-QUE 1 < PZQ-QUE 2 < PZQ-MYR. Considering instead the FT-IR analysis, the main differences were observed in the wavenumbers above 3000 cm^−1^: raw flavonols contain in their structures crystal water which interacts with their hydroxyl groups; after the formation of cocrystals with PZQ, no crystal water exists due to the interaction between flavonol hydroxyl groups and PZQ carbonyl groups, and therefore peaks in the region of 3000 cm^−1^ become weak.

Finally, as both flavonols and PZQ belong to BCS class II, flavonols being less soluble than PZQ, solubility studies were also carried out. The results showed that the solubility of the compound with higher solubility (i.e., PZQ) was reduced after the formation of the cocrystals, while the solubilities of the compounds with poor solubility (i.e., flavonols) were increased approximately three-fold.

Using the knowledge gained from the previous eight PZQ cocrystal structures deposited in the CSD, Devogelaer and coauthors used a network-based link-prediction algorithm [124,125] to predict 30 new coformer candidates for PZQ [70]. The list included very different compounds, such as five aliphatic dicarboxylic acids (four of which were previously considered by Espinosa-Lara et al.), benzoic acids derivatives, and aromatic compounds with hydroxyl, amine, and nitro groups. Also, salicylic acid and an enantiomer of tartaric acid were present in the list, the crystal structures of which were not previously solved [39]. Interestingly, 1,2,4,5-tetrafluoro-3,6-di-iodobenzene (DIFTB) was also predicted as a possible coformer for PZQ, even though its structure is very dissimilar to the other expected and reported coformers for PZQ. From these 30 coformers, 17 experimental indications for cocrystals were obtained but only 12 out of 17 were successfully grown as single crystals and analyzed through SXRD. Specifically, eight cocrystals were binary systems (i.e., PZQ-1,2,4,5-tetrafluoro-3,6-di-iodobenzene (PZQ-DITFB), PZQ-4-hydroxybenzoic acid (PZQ-4-HA), PZQ-3,5-dinitrobenzoic acid (PZQ-3,5-DNA), PZQ-hydroquinone (PZQ-HQ), PZQ-vanillic acid (PZQ-VA), PZQ-2,5-dihydroxybenzoic acid (PZQ-2,5-DHA), PZQ-2,4-hydroxybenzoic acid (PZQ-2,4-HA), and PZQ-orcinol (PZQ-ORC)), and four were cocrystal solvates (i.e., PZQ-salicylic acid hydrate (PZQ-SA MH), PZQ-4-aminosalicylic acid acetonitrile solvate (PZQ-4-ASA ACN), PZQ-2,5-dihydroxybenzoic acid acetonitrile solvate (PZQ-2,5-DHA ACN), and PZQ-3,5-dihydroxybenzoic acid acetonitrile solvate (PZQ-3,5-DHA ACN)). In all 12 crystal structures, PZQ molecules displayed the *anti*-conformation with the carbonyl groups in opposite directions and—in some cases—with a 90° rotation of the cyclohexyl ring. Moreover, all the crystal structures were centrosymmetric, containing therefore both the (R)- and the (S)-enantiomer of PZQ. Based on the intermolecular interaction patterns and the packing of PZQ enantiomers, the 12 crystal structures were organized into four different classes. The first class included PZQ-2,5-DHA, PZQ-2,5-DHA ACN, PZQ-4-HA, and PZQ-4-ASA ACN (refcodes in the CSD AVEHUH, AVEHIV, AVEJIX, and AVEJOD, respectively), which were characterized by one-dimensional enantiopure chains where both carbonyl groups of PZQ interacted with the coformer through H-bonds. As the crystal structures are centrosymmetric, they also contain chains of opposite chirality. The two cocrystal solvates of the class were isostructural, and their enantiopure chains lied in the same crystallographic direction ([111]). In case of PZQ-ORC (indexed as AVEHOB in the CSD), zigzag chains were visible and chains with an equal chirality stacked on top of each other. The second class comprised PZQ-HQ, PZQ-2,4-HA, and PZQ-3,5-DHA ACN (refcodes AVEKIJ, AVEJUJ, and AVEKEU). In this case, H-bonding patterns induced the formation of chains containing both the enantiomers of PZQ (1:1 molar ratio) and the coformer. Like the first class, both carbonyl groups of the alternating PZQ enantiomers took part in H-bond interaction with the coformer. On the contrary, for PZQ-VA, PZQ-3,5-DNA, and PZQ-SA MH (refcodes AVEJAP, AVEJET, and AVEHER), a class of so-called racemic pair cocrystals was identified where the (R)- and (S)-enantiomers of PZQ interact via H-bonds similarly to PZQ polymorph B. The fourth class just enclosed PZQ-DITFB (indexed as AVEKAQ in the CSD), which was the only molecule for which H-bonds were precluded, leaving halogen bonds and π–π interactions as plausible alternatives for cocrystal formation. Specifically, alternating PZQ enantiomers interact via carbonyl–iodide interactions, with the coformer and the fluorine atoms of the coformers additionally interacting with the hydrogens of the aromatic and cyclohexyl rings of PZQ. 

In a subsequent work, the same group focused attention on conducting a thorough comparison of the results obtained from the screening methods LAG, solvent evaporation (SE), and saturation temperature measurements (STM) (Figure 9) to review their advantages and drawbacks in cocrystal preparation [126]. LAG, SE, and STM enabled the identification of the previously discussed 17 cocrystals, with 14 showing stability and 12 new crystal structures solved. Going into detail, LAG experiments were performed by grinding a 1:1 molar ratio of PZQ and each coformer in the presence of reagent grade MeOH, EtOH, isopropanol (IPOH), AcT, and EA, and the output of the experiments was assessed by means of PXRD analysis. With LAG experiments, 11 coformers out of 30, namely 3,5-DNA, SA, DITFB, 4-HA, 4-ASA, HQ, VA, 2,5-DHA, 3,5-DNA, 2,4-DHA, and ORC, gave a new pattern not presenting traces of the starting materials. In most cases, systems screened with LAG in multiple solvents resulted in the same solid phase formation. Two coformers, i.e., VA and 2,5-DHA, gave, instead, two different PXRD patterns, depending on the solvent used. Therefore, 13 new systems for 11 positive coformers were identified through LAG experiments. Clarifications about the nature of the systems (i.e., cocrystals, cocrystal solvates, or cocrystal polymorphs) and their stoichiometry were given by the SXRD analysis of the crystal structures. Among the 13 new PXRD patterns, single-crystal growth experiments confirmed 12 new cocrystal structures where the simulated patterns corresponded to those obtained experimentally. Eight coformers gave 1:1 cocrystals with PZQ: DITFB, 4-HA, 4-ASA, HQ, VA, 2,5-DHA, 2,4-DHA, and ORC. Four cocrystals out of twelve were cocrystal solvates, with three of these being solvates with ACN (i.e., 4-ASA, 2,5-DHA, and 3,5-DHA). The fourth was a cocrystal hydrate unexpectedly obtained with SA through LAG in the presence of AcT, where water molecules were an impurity kept from ambient humidity. Interestingly, VA remained the unique semi-unclarified coformer: as above-mentioned, VA gave two different PXRD patterns if either using EtOH or ACN through LAG. The phase produced by using EtOH was a 1:1 cocrystal, whose structure was solved by SXRD. The other phase formed with ACN was not obtained through single-crystal growth experiments and therefore remained a question mark. 

SE experiments were performed with the same solvents of LAG to make comparison consistent. Ten coformers out of thirty (i.e., 3,5-DNA, PIM, SA, DITFB, 4-HA, HQ, VA, 2,5-DHA, 3,5-DHA, and 2,4-DHA) gave a new pattern not presenting traces of starting materials. Even in case of SE, 12 new PXRD patterns for 10 positive coformers were identified, and, as LAG outcomes, VA and 2,5-DHA gave two different PXRD patterns, depending on the solvent used. Worthy of notice is the fact that PXRD patterns obtained for HQ and 2,4-DHA through SE were not the same 1:1 cocrystals obtained by means of LAG, for which single crystals were grown. On the contrary, no single crystals were obtained for SE phases and the same problem was also observed for PIM, even though a new PXRD pattern was identified through SE experiments. For the other coformers, the PXRD patterns of SE products corresponded to those of LAG, except for 4-ASA, which did not give any cocrystal by means of SE, differing from LAG. 

For STM, 9 coformers out of 30 (i.e., 3,5-DNA, DITFB, 4-HA, 4-ASA, HQ, VA, 2,5-DHA, 3,5-DHA, and 2,4-DHA) showed a positive response in cocrystallization. A false positive was observed for benzoic acid in EtOH, as PXRD confirmed a physical mixture of PZQ and benzoic acid. In total, 12 new PXRD patterns for 9 positive coformers were obtained. As for LAG and SE, VA and 2,5-DHA gave two different outcomes due to the solvent used. The same was observed for 2,4-DHA, whose new pattern obtained in EtOH was specific to STM. However, no single crystal was grown in this case, so it was unclear whether it was a cocrystal, a polymorph, or a cocrystal solvate. For all other positive coformers, PXRD patterns were consistent with those obtained by LAG. 

To summarize, 12 new cocrystals were analyzed by means of SXRD and deposited in the CSD. All of them were identified with LAG, being the one found for ORC specific to LAG. For the VA coformer, two different patterns were obtained by using LAG, SE, and STM. The first was consistent with a 1:1 cocrystal, the crystal structure of which was solved through PXRD; the second remained an unresolved new phase, even though the possibility of a cocrystal solvate was excluded as the same result was obtained in several solvents. Presumably, this questioned phase could have been a cocrystal with a stoichiometry of 2:1 VA:PZQ, as the STM method required an excess of VA to obtain the experiments. Therefore, 13 stable cocrystals were discovered through LAG. With SE, 12 new systems were identified, with 9 in common with LAG and STM. The other three were specific to SE (PIM, HQ, and 2,4-DHA) and considered metastable due to inconsistency with LAG and STM experiments in the same conditions. As for STM, 12 new PXRD patterns were noticed, with 11 in common with LAG. A second cocrystal was obtained for 2,4-DHA in EtOH, being a specific result of STM. 

In summary, LAG was identified as the best, quickest, and most efficient screening route for cocrystallization, followed by STM and SE.

In the same year, Liu and coworkers reported a work aiming at investigating the solubility of four PZQ-carboxylic acids cocrystals and understanding their structural features by exploring the intermolecular weak interactions through theoretical calculations [71]. Citric acid (CA), phtalic acid (PA), 3-hydroxybenzoic acid (3-HA), and 4-hydroxybenzoic acid (4-HA) were chosen as coformers due to their good solubility and ground with PZQ by means of LAG to achieve cocrystallization (i.e., PZQ-CA, PZQ-PA, PZQ-3-HA, and PZQ-4-HA) (Figure 9). PZQ-CA was previously obtained, but its crystal structure was not solved [39]; the single crystal structure for PZQ-4-HA was obtained in a previous article but other characterization and solubility experiments were not performed [70]; PZQ-PA and PZQ-3-HA were previously predicted to form [70] but have never been obtained before.

The four cocrystals were fully characterized and the interactions between PZQ and each coformer were analyzed through theoretical calculations, namely atoms in molecules (AIM) topology analysis, electron density difference analysis (EDD), and energy decomposition analysis (EDA). 

PXRD patterns of the new products showed characteristic reflections not attributable to the starting materials, suggesting a 1:1 stoichiometry for PZQ-CA, PZQ-PA, and PZQ-4-HA, whereas a 2:1 molar ratio was suggested for PZQ-3-HA. Stoichiometries were confirmed by means of SXRD through which the crystal structure solution was possible. Precisely, PZQ-CA (refcode DAJYUM in the CSD) crystallizes in a 1:1 molar ratio in the orthorhombic space group of P2_1_2_1_2_1_; PZQ-PA (refcode DAJZIB) in the P2_1_/*c* space group of the monoclinic system; PZQ-3-HA and PZQ-4-HA (indexed as DAJZEX and AVEJIX01, respectively) crystallize in the triclinic space group of P-1. In all systems, PZQ carbonyl groups displayed an *anti*-conformation and SXRD analysis revealed that the main interactions between PZQ and coformers were H-bonds between PZQ carbonyl groups and coformer hydroxyl groups in different interaction modes. This evidence was also confirmed by FT-IR results in which shifts at lower frequencies or the disappearance of -OH stretching bands of coformers and shifts at higher frequencies for C=O stretching of PZQ were noticed. DSC analysis revealed the melting points for the four systems: the melting peak of PZQ-CA was detected at 137.2 °C, being lower than those of the starting materials and thus suggesting a worse thermal stability; the same was observed for PZQ-3-HA, which showed a melting point at 109.3 °C. On the contrary, PZQ-PA and PZQ-4-HA presented melting events at 150.0 °C and 155.0 °C, respectively, being in between the two pure components and demonstrating a better thermal stability compared to pure PZQ. 

Theoretical calculation methods such as AIM and EDD revealed the existence of classical and nonclassical H-bond interactions: classical O-H···O H-bond interactions were confirmed to be present in the structures, but also nonclassical interactions such as C-H···O were found, even if their strength was lower based on the EDD. EDA was then used to determine all the minor interactions present in the four systems and to clarify which of them mainly contributed to the formation of the cocrystal: H-bond interactions were demonstrated to be the main contributor, whereas forces such as dispersion and induction were relatively minor even though they could not be completely ignored. 

Furthermore, in vitro solubility tests in four media with different pH values were carried out and the results showed improved solubilities of all the cocrystals compared to pristine PZQ: after 4 h, the cocrystals’ solubility was about 4 times higher in the pH 1.2 medium, roughly 2 times higher in the pH 4.5 medium and water, and about 3.3 times higher in the pH 6.8 medium. PZQ-CA solubility was the worst of the four systems. 

In 2022, the same scientific group also reported PZQ cocrystallization in the presence of polyhydroxy phenolic acids, namely protocathecuic acid (PA), gallic acid (GA), and ferulic acid (FA) [72]. They prepared five different cocrystals through LAG and SE methods: precisely, PZQ-PA, PZQ-GA, and PZQ-FA were obtained by grinding in a mortar a 1:1 molar ratio of PZQ and each coformer in the presence of a certain amount of ACN (Figure 9); SE, performed with the same solvent, facilitated obtaining two cocrystal solvates, i.e., PZQ-PA-ACN and PZQ-GA-ACN, plus the same PZQ-FA cocrystal of LAG. These five new cocrystals were deeply characterized at the solid state, and the crystal structures of PZQ-PA-CAN, PZQ-GA-ACN, and PZQ-FA were solved. 

Unique endothermic peaks were visible in DSC curves for the three anhydrous cocrystals, and their melting points were in between those of PZQ and each coformer; the two cocrystal solvates, instead, showed endothermic events at 86.16 °C and 104.88 °C for PZQ-PA-ACN and PZQ-GA-ACN, respectively, corresponding to desolvation, as attested to also by TGA results. No further endothermic events were observed after desolvation, attesting that the crystalline lattice collapses after the loss of crystal ACN. 

PXRD analyses of the five cocrystals showed different patterns compared to those of PZQ and the coformers. Interestingly, the PZQ-PA and PZQ-GA patterns were similar, and the same behavior was observed also for PZQ-PA-ACN and PZQ-GA-ACN: the explanation lies in the very similar PA and GA structures that only differ in the five-position hydroxyl group. 

As the SE method enabled access to single crystals, three out of five cocrystals were analyzed by SXRD and their crystal structures were deposited in the CSD with deposition numbers of 2133511, 2133510, and 2133509 for PZQ-PA-ACN, PZQ-GA-ACN, and PZQ-FA, respectively. Both cocrystal solvates belong to the monoclinic I2/*a* space group and their ASU contains one PZQ, one PA/GA, and one solvent molecule, confirming a 1:1:1 PZQ:coformer:solvent stoichiometry. PZQ-FA belongs, instead, to the monoclinic space group of P2_1_/*n* and its ASU presents one PZQ and one FA molecule. In the two cocrystal solvates, phenolic acid forms H-bonds with PZQ and the solvent in the form of a monomer, while in PZQ-FA, phenolic acid first forms dimer and then interacts with PZQ by H-bonding. All three cocrystals displayed an *anti*-conformation of carbonyls of PZQ molecules.

Also, FT-IR analyses confirmed H-bond interactions and therefore cocrystal formation. Precisely, the interaction was assessed by the C=O stretching shift of carboxylic acids: in the case of cocrystal solvates, the characteristic vibration of the conjugated carboxylic acid monomer was found at 1716 cm^−1^, while the characteristic absorption peaks belonging to C=O stretching vibration of conjugated carboxylic acid dimer were observed at 1667, 1686, and 1686 cm^−1^ for PZQ-PA, PZQ-GA, and PZQ-FA, respectively. 

The mechanism of cocrystal formation was also discussed via theoretical calculations, including molecular interaction energy, EDD, and MEPS. 

Regarding the interaction energies of the cocrystal solvates, PZQ-PA-ACN exhibited lower bonding energy than PZQ-GA-ACN and the same was observed for the energies between PA and ACN and GA and ACN, supporting the much lower desolvation temperature of PZQ-PA-ACN compared to PZQ-GA-ACN. In the PZQ-FA, the interaction energy was the lowest, attesting that the connection between the FA dimer was the most stable and easy to generate. 

EDD and MEPS highlighted a decreasing electron density around hydroxyl oxygen atoms of coformers and an increasing electron density around the carbonyl oxygen of PZQ and, in case of cocrystal solvates, cyano nitrogen atoms of ACN, confirming the formation of H-bonds. 

Passing to biopharmaceutic properties, anhydrous cocrystals were selected to carry out solubility evaluations. Four different media (i.e., aqueous HCl solution (pH = 1.2), acetate buffer (pH = 4.5), phosphate buffer (pH = 6.8), and water) were used. Except for PZQ-PA in acetate buffer, the dissolution results in the four media differ remarkably from that of PZQ, and the cocrystals’ solubility was better. Based on the improvement of the solubility, PZQ-FA was selected as representative to also evaluate the biological activity in vivo. The Tmax and Cmax of PZQ and PZQ-FA were basically the same, but the absorption degree of the latter was superior. 

Recently, Yang et al. reported another work with the application of artificial intelligence in cocrystal screening [127]. Precisely, they provided a data-driven cocrystal prediction method based on the eXtreme Gradient Boosting (XGBoost) machine learning model of the scikit-learning package applied to their eight previously and experimentally obtained PZQ cocrystals [69,71,72]. The cocrystal data in the CSD and the data recorded as no cocrystal formation in experimental screening were used as data sets for model training. The structures of the drug and the coformers were represented by simplified molecular input line entry specification (SMILES) strings. RDkit molecular descriptors computed from the input SMILES strings were used as the features of the corresponding compound, which were computed by the ChemDes website [128]. This model, applied to PZQ cocrystallization, predicted that PZQ could form cocrystals with all eight coformers, which was consistent with experimental results [69,71,72], thus revealing the model as a powerful tool for cocrystal prediction and design in the field of drug research.

Coming back to experimentally discovered PZQ cocrystals, D’Abbrunzo and coworkers recently reported a drug–drug antiparasitic cocrystal presenting a very peculiar stoichiometry, almost unusual in the variety of cocrystals known in the literature. Precisely, the novel cocrystal was obtained by grinding PZQ and Niclosamide (NCM) in a 1:3 molar ratio in the presence of a catalytic amount of MeOH for 120 min at 25 Hz [73]. 

**Figure 10:**
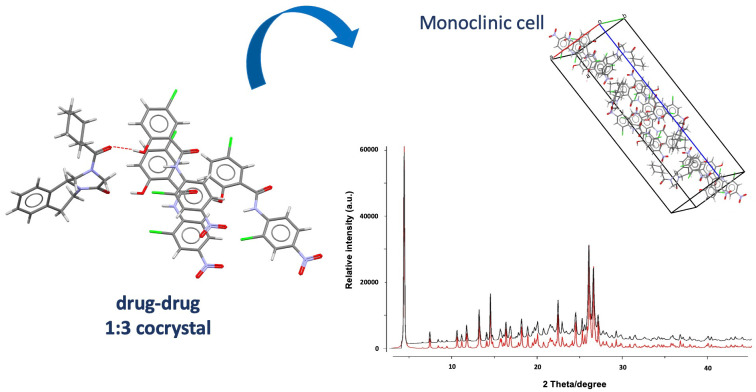
ASU (**left**), PXRD simulated from the single crystal and experimental powder patterns (**bottom right**), and crystal packing (**top right**). Adapted from reference [73].

SEM images showed that the new cocrystal consisted of agglomerates of small plates whose particle size roughly varies in the range of 150–1500 nm (see Figure 4). The DSC curve only showed an endothermic peak at 202.89 °C, attributable to the PZQ-NCM melting point and intermediate to those of pure PZQ and NCM (141.99 and 229.98 °C, respectively [59,129]). In addition, the laboratory diffraction pattern of the PZQ-NCM cocrystal showed reflections clearly different from those of the starting materials and in good agreement with the PXRD simulated from single crystals. The latter were obtained through conventional solution crystallization in EA starting from preformed seeds of PZQ-NCM cocrystal and analyzed through Synchrotron X-ray diffraction. The new cocrystal (indexed as RIPFOP01 in the CSD) crystallizes in the monoclinic unit cell with a space group of P2_1_/*c*, showing one PZQ and three NCM crystallographic independent molecules in the ASU (Figure 10). The centrosymmetric crystal packing is consistent with the presence of racemic PZQ, which is also partially disordered, in the unit cell. Structural analysis revealed that PZQ molecules exhibit a perpendicular relative orientation with respect to NCM molecules and all NCM molecules adopt a rigid, extended, and planar conformation defined by central amidic bond planarity constrain and homomolecular H-bonds in the crystal packing. The two carbonyl groups of PZQ act as H-acceptors bound to NCM donor hydroxyl groups and the third NCM present in the ASU is linked to the carbonyl of one NCM bound to PZQ. Interestingly, as in case of PZQ Form A, both PZQ carbonyl groups are oriented in the *syn* conformation, contrary to most of PZQ multicomponent systems discussed in this review. Also, a low-temperature crystal structure was deposited in the CSD and indexed as RIPFOP.

Intermolecular interactions and stoichiometry were also confirmed through FT-IR and SSNMR analyses. Considering the FT-IR results, the N-H stretching and bending peaks of NCM, originally at 3241–3101 cm^−1^ and 897 cm^−1^, respectively [130], were downward shifted, suggesting the involvement of the NCM N-H group in H-bonds with PZQ. Moreover, the carbonyl stretching vibration of PZQ at 1647 cm^−1^ was markedly shifted in the cocrystal, forming a doublet at 1694 and 1684 cm^−1^. Further, the signal of NCM C-OH stretching could be seen at 1231 cm^−1^, shifted at higher intensities in comparison to the raw peak at 1219 cm^−1^ [131].

Passing to the ^13^C CPMAS SSNMR spectrum, no traces of unreacted starting materials or PZQ polymorphs were detected, and a new crystalline phase was observed. Two isolated and split signals at about 153 and 175.6 ppm, respectively attributable to NCM and PZQ, were used to evaluate the stoichiometry ratio of the cocrystal: the integration of these two signals agreed with the presence of one PZQ and three NCM per ASU. In the ^15^N CPMAS spectrum, the N amide and nitro group chemical shifts demonstrated the formation of supramolecular interactions between PZQ and NCM and a new crystalline packing.

Also, the physical and chemical stability of PZQ-NCM were investigated under several conditions. As in previous works, a diminished PZQ recovery was noticed with the insurgence of peculiar degradation products as a function of the excipient used in binary ground systems [33,50,52], and the chemical stability of the cocrystal was examined through spectrometric evaluations: no typical PZQ tendency of decay was noticed in the experimental grinding conditions. 

Physical stability was evaluated both at the solid state and in aqueous solution. The PZQ-NCM cocrystal remained unchanged over a period of 12 months at ambient temperature with no signs of dissociation into the parent compounds. In the context of physical stability in aqueous solution, thanks to the strong supramolecular interactions, the cocrystal structure confers a resistance to the otherwise predominant transition of NCM into the insoluble and undesired monohydrate NCM Ha, which usually arises within 1 month of NCM storage at ambient temperature [129].

More importantly, the PZQ-NCM cocrystal exhibited higher anthelmintic activity (%-effect of activity reduction) against in vitro adult *Schistosoma mansoni* models compared to the corresponding physical mixture (PM). Based on these promising results, in vivo preliminary tests were also carried out: the new solid was administered as a powder in minicapsule size M (specific for mice) instead of the conventional aqueous suspensions commonly used during in vivo administration [94]. Despite the limited number of mice treated, there was no significant difference between the number of worms recovered from infected mice treated with PZQ-NCM cocrystal and those with PM. In the case of treatment with pure PZQ, a comparison with the cocrystal was ineffective due to an underdosage compared to PZQ monotherapy. However, the administration of pure PZQ to mice resulted a positive control group, since it confirmed that the treatment with minicapsules worked, revealing cocrystal higher doses encouraging for future in vivo studies.

A different approach to cocrystal screening protocols was reported by Cappuccino et al., who, instead of searching for other coformers for PZQ cocrystallization, investigated cocrystalline solid solution formation of PZQ in the presence of enantiomerically pure malic (MAL) and tartaric (TA) acids in scalemic and racemic stoichiometry [74]. Two new cocrystals were structurally characterized and deposited in the CSD and three non-stoichiometric mixed crystal forms were identified and isolated. Based on a previous work that reported the 1:1 cocrystallization of (RS)-PZQ in the presence of enantiomerically pure L-MAL, which allowed the resolution of two diastereomeric cocrystals (i.e., (R)-PZQ/L-MAL and (S)-PZQ/L-MAL) [68], in this work the authors investigated the crystallization of PZQ in the presence of racemic MA and obtained a four-component new cocrystal, (R)-PZQ/(S)-PZQ/D-MAL/L-MAL. Its crystal structure (CCDC deposition number 2205816) was solved by PXRD, as no single crystals were grown. PZQ and MAL molecules alternate into racemic H-bonded chains along the b axis of the orthorhombic *Pbca* unit cell, with the acidic oxygen of the carboxylic acid bridging between two different carbonyl oxygen atoms of two racemic PZQs. The hydroxyl group of MAL acts as an H-bond donor in an intramolecular H-bond for the adjacent carboxylic group, while it acts as an H-bond acceptor in an additional H-bond with another MAL from the nearest chains aligned in an antiparallel orientation in the crystal. PZQ carbonyl groups displayed the most common *anti*-conformation.

A similar four-component phase was obtained by grinding PZQ in the presence of racemic TA (i.e., (R)-PZQ/(S)-PZQ/D-TA/L-TA). In this crystal structure (CCDC deposition number 2205817), chains of (S)-PZQ and L-TA extend along an axis of the triclinic P-1 unit cell and alternate with homologous chains of (R)-PZQ and D-TA kept together by the same monodentate interaction between the TA acidic group and the PZQ carbonyl group. Adjacent homochiral chains are held together by intermolecular H-bonds involving hydroxyl groups. Even in this case, PZQ molecules presented an *anti*-conformation. 

In case of using TA, no structure was determined from the mechanochemical crystallization of (RS)-PZQ in the presence of D-TA, so it remained unclear whether the system was a three-component system (i.e., (R)-PZQ/(S)-PZQ/D-TA) or a mixture of diastereomeric crystals (i.e., (R)-PZQ/D-TA and (S)-PZQ/D-TA), as for MAL. 

Subsequently, the authors tried scalemic mixtures and substitutions between MAL and TA to assess the formation of (RS)-PZQ solid solutions with varied acid compositions. 

Precisely, milling (RS)-PZQ in the presence of scalemic ratios of MAL (i.e., (R)-PZQ/(S)-PZQ/(D)-MAL/(L)-MAL = 1.5:1.5:1:2) did not give (R)-PZQ/L-MAL and (S)-PZQ/L-MAL, whereas it formed (R)-PZQ/(S)-PZQ/D-MAL/L-MAL, pointing toward the formation of a solid solution. DSC curves showed, indeed, a superimposition between the peak observed for (R)-PZQ/(S)-PZQ/D-MAL/L-MAL and the obtained solid solution. 

A similar result was observed in the case of TA: grinding (RS)-PZQ in the presence of D-TA and L-TA in a 3:1:2 ratio (i.e., (R)-PZQ/(S)-PZQ/(D)-TA/(L)-TA = 1.5:1.5:1:2) gave (R)-PZQ/(S)-PZQ/D-TA/L-TA, as also confirmed by DSC results. 

Moreover, the LAG of (RS)-PZQ with homochiral D-TA and L-MAL in a 1.5:1.5:1:2 ratio did not provide a solid solution but gave a mixture of (R)-PZQ/L-MAL and (S)-PZQ/L-MAL and the above-mentioned unknown product with D-TA.

When (RS)-PZQ was milled in the presence of the pseudoracemate (1:1 mixture) of L-MAL and L-TA, the unknown product with D-TA was obtained and the same was observed even by increasing the amount of L-TA compared to that of L-MAL, suggesting that a four-component cocrystalline solid solution is obtained at a high TA content. (R)-PZQ/L-MAL and (S)-PZQ/L-MAL only appeared at the higher ratio of L-MAL. 

The authors also highlighted the possibility of obtaining a five-component solid solution by substituting part of L-MAL with D-MAL in the unknown product with D-TA (to obtain (R)-PZQ/(S)-PZQ/D-MAL/L-MAL/L-TA) or part of D-TA with L-MAL in (R)-PZQ/(S)-PZQ/D-TA/L-TA (to obtain (R)-PZQ/(S)-PZQ/L-MAL/D-TA/L-TA). Instead, physical mixtures were obtained while attempting the substitution of D-MAL with L-TA in the cocrystal (R)-PZQ/(S)-PZQ/D-MAL/L-MAL (to obtain (R)-PZQ/(S)-PZQ/D-MAL/L-MAL/L-TA) and the solid solution with the six components together. 

Furthermore, solubility analysis indicated a four-fold solubility advantage for the newly prepared solid solutions over the pure drug and a faster dissolution rate, probably due to the metastable character of these solid solutions. To evaluate whether the increase in solubility and dissolution rate translated into higher oral bioavailability, in vivo pilot tests were performed on rats by administering solid solutions as powder for the first time in minicapsule size nine. Solid solutions demonstrated a faster absorption compared to the pure drug and helped to maintain a constant steady-state concentration.

## 7. Conclusions

Praziquantel has always been the recommended drug against all species of schistosomiasis. It is included in the WHO Model List of Essential Drugs for the treatment of both adults and children and has been on the market for 50 years. Yet, only recently has it attracted more attention, namely in the past decade, with a renewed wave of interest (i.e., novel solid forms). The explanation of this phenomenon lies in the fact that praziquantel can exhibit a variety of structures with different inter- and intra-molecular interactions as well as a distinct propensity to form multicomponent crystals (i.e., interacting with water, solvents, and structurally different coformers). Progress in crystal engineering science (e.g., the use of mechanochemistry as a solid form screening tool and more strategic structure-based methods) and the development of analytical techniques, including Synchrotron X-ray analyses, spectroscopy, and microscopy, have expediated the identification of unknown crystal structures of the drug. Also, computational modeling has given a significant contribution to the prediction and designing of new cocrystals via structural conformations and interactions energy analyses. 

To date, the literature provides 7 anhydrous polymorphs (counting the commercially available Form A), 6 hydrates, 3 solvates, and 44 cocrystals, including 1 cocrystal monohydrate and 5 cocrystal solvates. 

Given the large number of solid forms of praziquantel found in just a few years, increasing attention is to be expected in the near future. Indeed, the higher the number of solid forms, the stronger the scientific interest in terms of experimental research and pharmaceutical applicability. Specifically, being familiar with praziquantel polymorphic forms might help in controlling their formation during manufacturing and drug formulation processes. Further, multicomponent systems such as PZQ-HH, having displayed significant improvement of praziquantel physicochemical and biopharmaceutical properties, will be relevant for further investigation from a pharmaceutical standpoint. In the cocrystal scenario, besides dicarboxylic acids (the coformers of choice by several authors), other considered coformers are not always found on the so-called GRAS (generally recognized as safe) list [132]. More recently, attention has been drawn to drug-like molecules as potential coformers (including flavonols), finally paving the way for the development of a proper drug-to-drug praziquantel cocrystal, and the pharmaceutical applicability thereof may be further investigated.

As a result, some questions arise almost spontaneously: Could praziquantel be the new sulfathiazole? Might the active substance be repositioned? And most importantly, might the research on new multicomponent forms of praziquantel with GRAS coformers, drug-like molecules, or other drugs solve the bitter taste barrier, low solubility, low bioavailability, and extensive first-pass metabolism, once and for all?

## Figures and Tables

**Table 1:** Reported solid forms for PZQ.

Acronym	Type of Solid Form	Stoichiometry	CSD Reference Code	References
	**Anhydrous polymorphs**			
PZQ Form A	polymorph A	N/A ^§^	TELCEU	[58]
PZQ Form B	polymorph B	N/A ^§^	TELCEU01	[59]
PZQ Form C	polymorph C	N/A ^§^	GOYZOM	[60]
PZQ Form D	polymorph D	N/A ^§^	not indexed	[61]
PZQ Form E	polymorph E	N/A ^§^	not indexed	[61]
PZQ Form G	polymorph G	N/A ^§^	not indexed	[62]
PZQ Form H	polymorph H	N/A ^§^	not indexed	[62]
	**Hydrates**			
(S)-PZQ-HH	(S)-praziquantel hemihydrate	1:0.5	SIGBUG	[25]
(R)-PZQ-HH	(R)-praziquantel hemihydrate	1:0.5	SIGBUG01	[26]
(R)-PZQ-MH	(R)-praziquantel monohydrate	1:1	LIVFED	[19]
PZQ-MH	praziquantel monohydrate	1:1	not indexed	[63]
PZQ-HH	(RS)-praziquantel hemihydrate	1:0.5	WUHQAU	[64]
PZQ-HH	(RS)-praziquantel hemihydrate	1:0.5	not indexed	[65]
	**Solvates**			
PZQ-2P	praziquantel-2-pyrrolidone	1:1	DAJCAW	[66]
PZQ-AA	praziquantel-acetic acid	1:1	DAJCEA	[66]
PZQ-DMA	praziquantel-dimethylacetamide	1:1	not indexed	[62]
	**Cocrystals**			
PZQ-OXA	praziquantel-oxalic acid	1:1	TELCOE	[58]
PZQ-MALO	praziquantel-malonic acid	1:1	TELDEV	[58]
α-PZQ-SUC	praziquantel-succinic acid Form α	1:1	not indexed	[58]
β-PZQ-SUC	praziquantel-succinic acid Form β	1:1	TELDAR	[58]
PZQ-MALE	praziquantel-maleic acid	1:1	TELCIY	[58]
PZQ-FUM	praziquantel-fumaric acid	1:1	TELBUJ	[58]
PZQ-GLU	praziquantel-glutaric acid	1:1	TELDIZ	[58]
PZQ-ADI	praziquantel-adipic acid	2:1	TELCAQ	[58]
PZQ-PIM	praziquantel-pimelic acid	2:1	not indexed	[58]
(R)-PZQ-GLU	(R)-praziquantel-glutaric acid	1:1	KEQVEL	[67]
(R)-PZQ-SUC	(R)-praziquantel-succinic acid	1:1	KEQVAH	[67]
(R)-PZQ/L-MAL	(R)-praziquantel-(L)-malic acid	1:1	CUZPIY	[68]
(S)-PZQ/L-MAL	(S)-praziquantel-(L)-malic acid	1:1	CUZPEU	[68]
PZQ-CA	praziquantel-citric acid	1:1	not indexed	[39]
PZQ-MAL	praziquantel-malic acid	1:1	not indexed	[39]
PZQ-SA	praziquantel-salicylic acid	1:1	not indexed	[39]
PZQ-TA	praziquantel-tartaric acid	1:1	not indexed	[39]
PZQ-KAE	praziquantel-kaempferol	2:1	ANAYOG	[69]
PZQ-QUE 1	praziquantel-quercetin 1	2:1	ANAYUM	[69]
PZQ-QUE 2	praziquantel-quercetin 2	1:1	not indexed	[69]
PZQ-MYR	praziquantel-myricetin	1:1	ANAZAT	[69]
PZQ-DITFB	praziquantel-1,2,4,5-tetrafluoro-3,6-di-iodobenzene	1:1	AVEKAQ	[70]
PZQ-4-HA	praziquantel-4-hydroxybenzoic acid	1:1	AVEJIX	[70]
PZQ-3,5-DNA	praziquantel-3,5-dinitrobenzoic acid	1:1	AVEJET	[70]
PZQ-HQ	praziquantel-hydroquinone	1:1	AVEKIJ	[70]
PZQ-VA	praziquantel-vanillic acid	1:1	AVEJAP	[70]
PZQ-VA	praziquantel-vanillic acid	1:2	not indexed	[70]
PZQ-2,5-DHA	praziquantel-2,5-dihydroxybenzoic acid	1:1	AVEHUH	[70]
PZQ-2,4-HA	praziquantel-2,4-dihydroxybenzoic acid	1:1	AVEJUJ	[70]
PZQ-ORC	praziquantel-orcinol	1:1	AVEHOB	[70]
PZQ-SA MH	praziquantel-salicylic acid monohydrate	1:1	AVEHER	[70]
PZQ-4-ASA ACN	praziquantel-4-aminosalicylic acid acetonitrile solvate	1:1:1	AVEJOD	[70]
PZQ-2,5-DHA ACN	praziquantel-2,5-dihydroxybenzoic acid acetonitrile solvate	1:1:1	AVEHIV	[70]
PZQ-3,5-DHA ACN	praziquantel-3,5-dihydroxybenzoic acid acetonitrile solvate	1:1:1	AVEKEU	[70]
PZQ-CA	praziquantel-citric acid	1:1	DAJYUM	[71]
PZQ-PA	praziquantel-phtalic acid	1:1	DAJZIB	[71]
PZQ-3-HA	praziquantel-3-hydroxybenzoic acid	2:1	DAJZEX	[71]
PZQ-4-HA	praziquantel-4-hydroxybenzoic acid	1:1	AVEJIX01	[71]
PZQ-PA	praziquantel-protocathecuic acid	1:1	not indexed	[72]
PZQ-GA	praziquantel-gallic acid	1:1	not indexed	[72]
PZQ-FA	praziquantel-ferulic acid	1:1	2133509 *	[72]
PZQ-PA-ACN	praziquantel-protocathecuic acid acetonitrile solvate	1:1:1	2133511 *	[72]
PZQ-GA-ACN	praziquantel-gallic acid acetonitrile solvate	1:1:1	2133510 *	[72]
PZQ-NCM	praziquantel-niclosamide	1:3	RIPFOP	[73]
(R)-PZQ/(S)-PZQ/D-MAL/L-MAL	(R)-praziquantel-(S)-praziquantel-(D)-malic acid-(L)-malic acid cocrystal	1:1:1:1	2205816 *	[74]
(R)-PZQ/(S)-PZQ/D-TA/L-TA	(R)-praziquantel-(S)-praziquantel-(D)-tartaric acid-(L)-tartaric acid cocrystal	1:1:1:1	2205817 *	[74]

^§^ not applicable for single compound. * CCDC (Cambridge Crystallographic Data Centre) deposition number.

## Abbreviations

The following abbreviations are used in this review:

AA	acetic acid
CAN	Acetonitrile
AcT	Acetone
AIM	atoms in molecules
API	active pharmaceutical ingredient
ASU	asymmetric unit
AUC	area under the curve
BCS	Biopharmaceutics Classification System
CCDC	Cambridge Crystallographic Data Centre
CHF	chloroform
CSD	Cambridge Structural Database
CyHXN	cyclohexanone
DFT	density functional theory
DMA	dimethylacetamide
DMSO	dimethyl sulfoxide
DSC	differential scanning calorimetry
EA	ethyl acetate
EDA	energy decomposition analysis
EDD	electron density difference
1,2-EtdOH	1,2-ethanediol
EtOH	ethanol
FE-SEM	field-emission SEM
FT-IR	Fourier Transform Infrared spectroscopy
GRAS	generally recognized as safe
H-bonds	hydrogen bonds
HPC	hydroxy propyl cellulose
HSM	hot-stage microscopy
n-HXN	n-hexane
H_2_O	water
IDR	intrinsic dissolution rate
IPOH	isopropanol
LAG	liquid-assisted grinding
MeOH	methanol
MEPS	molecular electrostatic potential surfaces
MTDSC	modulated differential scanning calorimetry
NG	neat grinding
PM	physical mixture
2-PROH	2-propanol
PXRD	powder X-ray diffraction
PZQ	Praziquantel
2-pyr	2-pyrrolidone
RH %	relative humidity percentage
SEE	solvent evaporation experiment
SEM	scanning electron microscopy
SI	saturation index
SPE	(Single Pulse Excitation)
SSNMR	solid-state NMR
STM	saturation temperature measurements
SXRD	single-crystal X-ray diffraction
TEA	triethylamine
TGA	thermogravimetric analysis
THF	tetrahydrofuran
WHO	World Health Organization
WBR	worm burden reduction
